# The wheat stem rust resistance gene *Sr43* encodes an unusual protein kinase

**DOI:** 10.1038/s41588-023-01402-1

**Published:** 2023-05-22

**Authors:** Guotai Yu, Oadi Matny, Spyridon Gourdoupis, Naganand Rayapuram, Fatimah R. Aljedaani, Yan L. Wang, Thorsten Nürnberger, Ryan Johnson, Emma E. Crean, Isabel M.-L. Saur, Catherine Gardener, Yajuan Yue, Ngonidzashe Kangara, Burkhard Steuernagel, Sadiye Hayta, Mark Smedley, Wendy Harwood, Mehran Patpour, Shuangye Wu, Jesse Poland, Jonathan D. G. Jones, T. Lynne Reuber, Moshe Ronen, Amir Sharon, Matthew N. Rouse, Steven Xu, Kateřina Holušová, Jan Bartoš, István Molnár, Miroslava Karafiátová, Heribert Hirt, Ikram Blilou, Łukasz Jaremko, Jaroslav Doležel, Brian J. Steffenson, Brande B. H. Wulff

**Affiliations:** 1 Plant Science Program, Biological and Environmental Science and Engineering Division (BESE), King Abdullah University of Science and Technology (KAUST), Thuwal, Saudi Arabia; 2 Center for Desert Agriculture, KAUST, Thuwal, Saudi Arabia; 3 John Innes Centre, Norwich Research Park, Norwich, UK; 4 Department of Plant Pathology, University of Minnesota, St. Paul, MN USA; 5 Bioscience Program, Smart Health Initiative, BESE, KAUST, Thuwal, Saudi Arabia; 6 Department of Plant Biochemistry, Centre of Plant Molecular Biology (ZMBP), University of Tübingen, Tübingen, Germany; 7 Institute for Plant Sciences, University of Cologne, Cologne, Germany; 8 Cluster of Excellence on Plant Sciences (CEPLAS), Cologne, Germany; 9 Department of Agroecology, Aarhus University, Slagelse, Denmark; 10 Department of Plant Pathology, Kansas State University, Manhattan, KS USA; 11 The Sainsbury Laboratory, University of East Anglia, Norwich, UK; 12 2Blades Foundation, Evanston, IL USA; 13 Institute for Cereal Crops Research, Tel Aviv University, Tel Aviv, Israel; 14 Institute for Cereal Crops Research, and the School of Plant Sciences and Food Security, Tel Aviv University, Tel Aviv, Israel; 15 USDA-ARS, Cereal Disease Laboratory, St. Paul, MN USA; 16 Crop Improvement and Genetics Research Unit, USDA-ARS, Western Regional Research Center, Albany, CA USA; 17 Centre of the Region Haná for Biotechnological and Agricultural Research, Institute of Experimental Botany of the Czech Academy of Sciences, Olomouc, Czech Republic; 18 Red Sea Research Center, BESE, KAUST, Thuwal, Saudi Arabia; 19 Present Address: Enko Chem, Mystic, CT USA; 20 Present Address: Centre for Agricultural Research, ELKH, Agricultural Institute, Martonvásár, Hungary

**Keywords:** Plant genetics, Plant breeding, Biotechnology, Plant molecular biology

## Abstract

To safeguard bread wheat against pests and diseases, breeders have introduced over 200 resistance genes into its genome, thus nearly doubling the number of designated resistance genes in the wheat gene pool^1^. Isolating these genes facilitates their fast-tracking in breeding programs and incorporation into polygene stacks for more durable resistance. We cloned the stem rust resistance gene *Sr43*, which was crossed into bread wheat from the wild grass *Thinopyrum elongatum*^2,3^. *Sr43* encodes an active protein kinase fused to two domains of unknown function. The gene, which is unique to the Triticeae, appears to have arisen through a gene fusion event 6.7 to 11.6 million years ago. Transgenic expression of *Sr43* in wheat conferred high levels of resistance to a wide range of isolates of the pathogen causing stem rust, highlighting the potential value of *Sr43* in resistance breeding and engineering.

## Abstract (web summary)

The resistance gene *Sr43*, which was crossed into bread wheat from the wild grass *Thinopyrum elongatum*, encodes an unusual protein kinase fusion protein that confers wheat stem rust resistance.

## Main

Worldwide, ~20% of projected bread wheat (*Triticum aestivum*) production is lost to pests and diseases every year^4^. The deployment of genetic variation for disease resistance is a sustainable and environmentally friendly way to protect wheat crops^5^. For over 100 years, breeders have conducted numerous crosses to enrich the wheat gene pool with resistance genes. Notably, more than 200 of the 467 currently designated resistance genes in cultivated bread wheat have their origin outside the bread wheat gene pool^1^. However, the deployment of these interspecific resistance genes is often hampered by linkage drag, that is, the cointroduction of deleterious alleles from linked genes. Moreover, single resistance genes tend to be rapidly overcome by the emergence of resistance-breaking pathogen strains^6^. Cloning individual resistance genes would enable their introduction as genetically modified polygene stacks, which are likely to provide more durable resistance^7^.

Most of the ~291 plant disease resistance genes cloned to date encode either intracellular receptors of the nucleotide-binding and leucine-rich repeat (NLR) class or extracellular membrane-anchored receptor-like proteins (RLPs, called RLKs when they contain an intracellular kinase) (Supplementary Table 1)^1,8^. A new group of resistance genes has recently come to light, whose members encode two protein kinases fused as one protein. These tandem kinase genes include *Rpg1*, *Yr15*, *Sr60*, *Sr62*, *Pm24*, *WTK4* and *Rwt4* (refs. ^9–15^). Other resistance genes offer some variation to this architecture with protein kinases fused to a steroidogenic acute regulatory protein-related lipid transfer domain (*Yr36*)^16^, a C2 domain and a multitransmembrane region (*Pm4*)^17^, a major sperm protein (*Snn3*)^18^, an NLR (*Tsn1*, *Rpg5* and *Sm1*)^19–21^ and a von Willebrand factor type A domain (*Lr9*)^22^.

These kinase fusion protein-encoding resistance genes appear to be unique to the Triticeae, the clade of grasses that arose 12 million years ago and encompasses the cereals wheat, rye (*Secale cereale*) and barley (*Hordeum vulgare*)^23^. However, the fusion events that gave rise to these genes, far from being rare and isolated, happened many times between different classes of kinases and spawned diverse combinations^10,12^. This genomic innovation resulted in resistance against phylogenetically distinct fungal pathogens spanning the ~300 million-year-old ascomycete/basidiomycete divide.

**Fig. 1:**
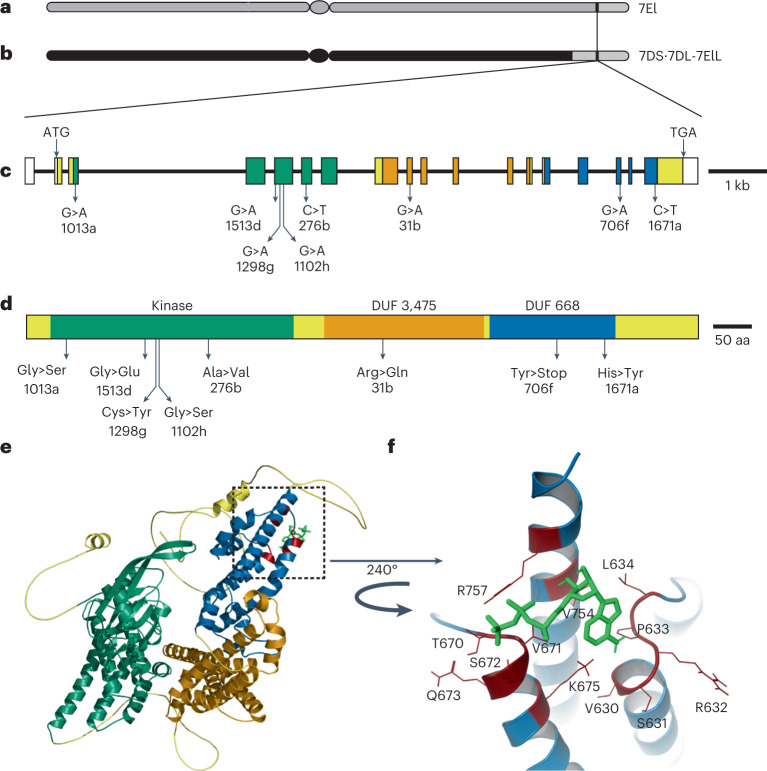
*Sr43* encodes a protein kinase fused to two DUFs. **a**, *Th. elongatum* chromosome 7. **b**, Schematic diagram of the wheat–*Thinopyrum* translocation chromosome. **c**, Identified EMS mutations along the predicted *Sr43* gene model. **d**, Schematic diagram of Sr43, showing predicted domains and amino acid changes induced by the EMS mutations. **e**, Three-dimensional model of Sr43, as predicted by AlphaFold. Green, kinase; orange and blue, DUF domains; yellow, flexible linkers. **f**, Structural detail (dashed box in **e**) of a high-confidence ATP-binding site (red residues) in DUF668 bound to an ATP molecule, as determined by the small molecule docking program HADDOCK^28^. ATP is depicted as a stick structure (light green) connected to DUF668 residues by hydrogen bonds (red lines).

Here, we cloned the stem rust resistance gene *Sr43*, which was transferred from tall wheatgrass (*Thinopyrum elongatum*) into bread wheat 45 years ago^2,3^. The dominant resistance gene *Sr43* was introgressed into chromosome 7D of hexaploid wheat (Fig. 1a,b). We mutagenized grains of the *Sr43* introgression line with ethyl methanesulfonate (EMS) and screened 2,244 surviving M_2_ families for susceptibility to *Puccinia graminis* f. sp. *tritici* (*Pgt*). We identified 23 families segregating for stem rust susceptibility, of which we confirmed ten independent mutants by progeny testing (Supplementary Table 2) and genotyping (Supplementary Figs. 1 to 11).

**Extended Data Fig. 1:**
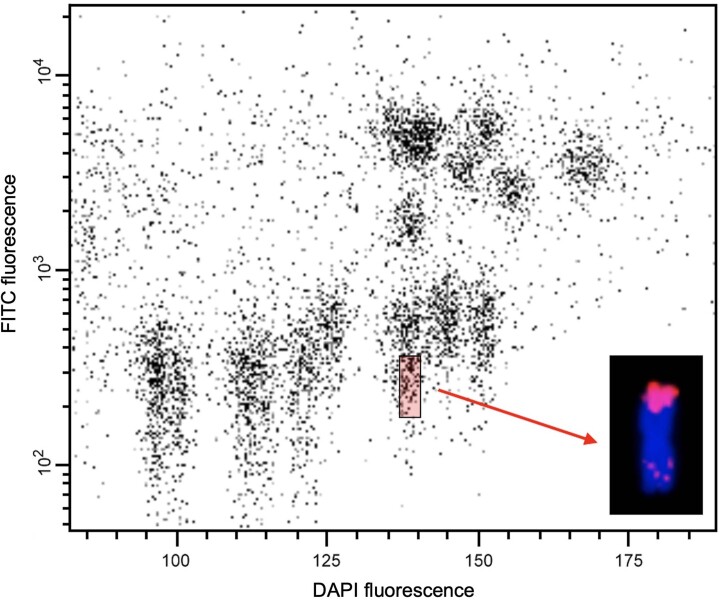
Bivariate flow karyotype of mitotic metaphase chromosomes isolated from the wheat-*Th. elongatum* 7D/7el2 translocation line carrying the *Sr43* resistance gene. DAPI (x-axis) vs. FITC (y-axis) dot plot was obtained after the analysis of DAPI-stained chromosome suspensions labeled by FISHIS with FITC-conjugated probes for GAA and ACG microsatellites. The 7D/7el2 translocation chromosomes were sorted from the sorting window shown as red rectangle at purities of 60–65%. Inset: 7D/7el2 translocation chromosome after FISH with probes for pSc119.2 repeat (green), Afa family repeat (red) and 45S rDNA (yellow) that was used to identify chromosomes in the sorted fraction. Chromosomes were counterstained by DAPI (blue).

**Extended Data Fig. 2:**
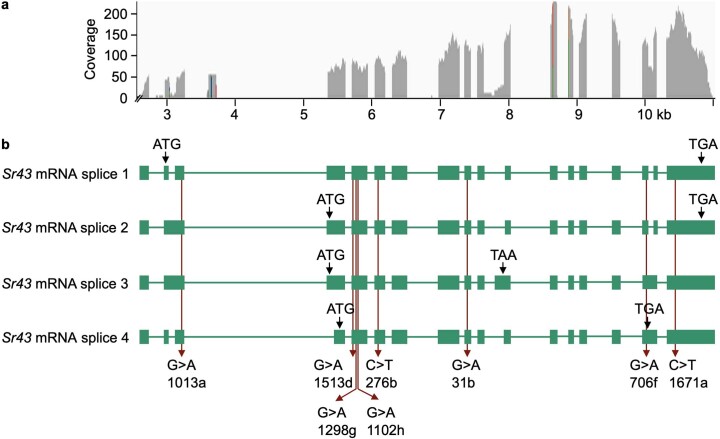
*Sr43* transcript and alternative splicing. **a**, Mapping of RNA-seq reads from the leaf transcriptome onto the *Sr43* locus. **b**, mRNA splice variants identified by sequencing full-length cDNA clones. Introns and exons are represented by lines and boxes, respectively. Splice variants 1, 2, 3 and 4 have 8, 7, 5 and 6 EMS-induced mutations in the coding sequence, respectively.

To clone *Sr43*, we performed chromosome flow sorting and sequenced the wheat–*Th. elongatum* recombinant chromosome 7D in the parental line and eight mutants (Extended Data Fig. 1 and Supplementary Tables 3 and 4). Sequence assembly of the parental line and mapping of the mutant reads identified a 10 kilobase (kb) window in a scaffold containing a mutation in all eight mutants. To determine the gene structure of the *Sr43* candidate, we (1) conducted transcriptome deep sequencing (RNA-seq) analysis of *Sr43* seedling leaves and mapped the reads to the *Sr43* genomic scaffold (Extended Data Fig. 2a) and (2) sequenced *Sr43* clones obtained by polymerase chain reaction (PCR) from a full-length complementary DNA library. We detected four different splice variants (Extended Data Fig. 2b and Supplementary Tables 5 and 6). Splice variant 1 contained all eight mutations and consisted of 18 exons with a predicted open reading frame of 2,598 base pairs (bp) (Fig. 1c). The eight mutations were all G/C to A/T transitions typical of EMS mutagenesis and introduced non-synonymous changes (seven mutants) or an early stop codon in the predicted coding sequence (Fig. 1c,d and Supplementary Tables 7 and 8). The probability that all mutants would have a mutation in the same gene by chance alone out of the 5,822 non-redundant genes of chromosome 7D (ref. ^24^) was 4 × 10^–6^, indicating that the identified gene is a good candidate for *Sr43*.

As all identified EMS mutations affected the predominant full-length *Sr43* transcript (Fig. 1c), we used its predicted 866–amino acid sequence to search for functional domains and homologs. We determined that Sr43 harbors an N-terminal kinase domain and two domains of unknown function (DUFs) in its C terminus (Fig. 1d). Five of the mutations resided within the kinase domain, with the remaining three mutations affecting either DUF (Fig. 1d).

**Extended Data Fig. 3:**
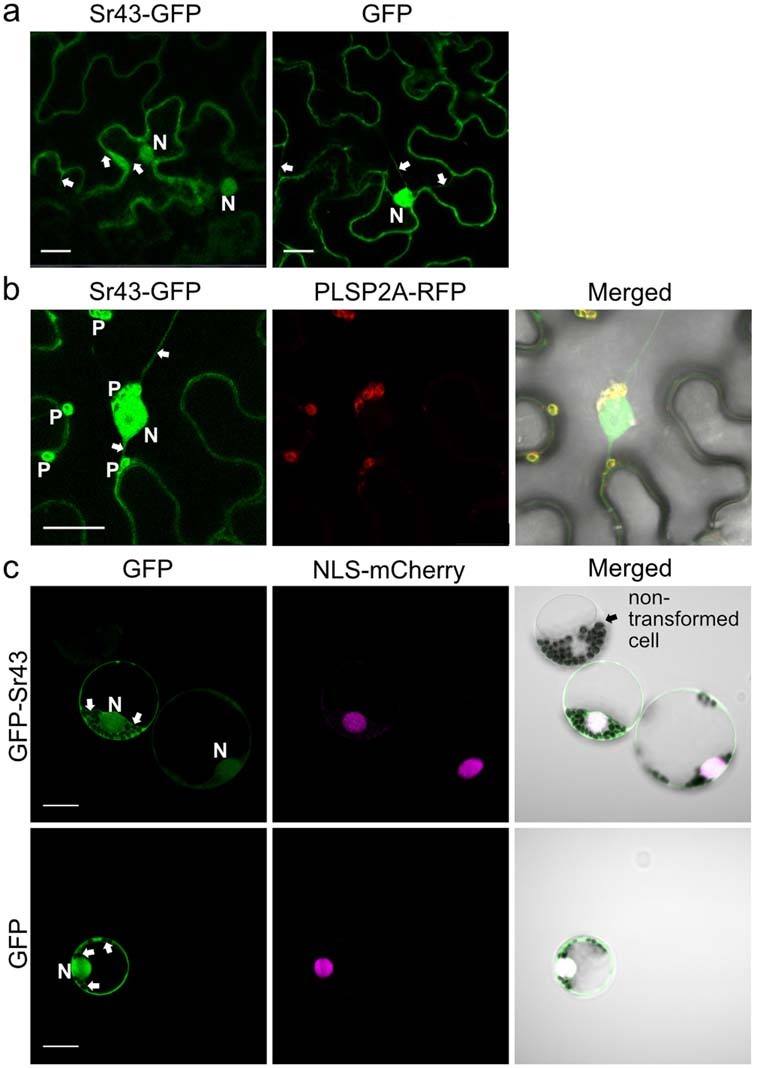
Sr43 localizes to the nucleus and plastids. **a**, *Agrobacterium*-mediated transient expression of a *Sr43-GFP* fusion construct or a *GFP* construct driven by the cauliflower mosaic virus (CaMV) 35 S promoter in *Nicotiana benthamiana*. GFP fluorescence in epidermal cells was recorded at 3 days post infiltration by confocal laser scanning microscopy. **b**, *Agrobacterium*-mediated transient expression of a the 35 S:*Sr43-GFP* with a 35 S:*PLSP2A-RFP* plastid marker construct in *N. benthamiana*. GFP or RFP fluorescence in epidermal cells was recorded at 3 days post infiltration by confocal laser scanning microscopy. **c**, transient expression of a ZmUbi:*GFP-Sr43* fusion construct with ZmUbi:*NLS-mCherry* nuclear marker in wheat mesophyll cells (upper panel) and a ZmUbi:*GFP* as control (lower panel). GFP and mCherry fluorescence were recorded 16–20 hrs post transfection by confocal laser scanning microscopy. A non-transformed cell is indicated with black arrow. The scale bar in all the images is 20 µm. Nuclei (N), cytoplasm (C) and plastids (P) are indicated on the images with white arrows. The sizes of the GFP and Sr43-GFP fusion proteins are estimated at 27 and 126 kDa, respectively. The experiments were repeated in five (Sr43-GFP, panel a), two (GFP, panel b), three (panel b), five (GFP-Sr43) and two (GFP) independent experiments, respectively, with similar results. In the *N. benthamiana* experiments, we infiltrated two leaves and screened more than 100 nuclei per leaf.

The closest BLAST homolog of the Sr43 kinase domain was the serine/threonine kinase interleukin-1 receptor-associated kinase (STKc IRAK) (Supplementary Fig. 12), indicating that Sr43 is probably a kinase. Further homology searches suggested that the kinase domain is intact (Supplementary Fig. 13). Supporting this observation, we found that an affinity-tagged Sr43 fusion protein purified from *Eschericia coli* phosphorylated maltose-binding protein DNA gyrase in vitro (Supplementary Figs. 14–16 and Supplementary Table 9). Moreover, mutant 1013a disrupted one of the conserved glycine residues in the glycine-rich loop, suggesting that kinase activity is required for Sr43 function (Supplementary Table 10). The C terminus of Sr43, containing DUF3475 and DUF668, has a similar domain architecture (44% identity) to the N terminus of PHYTOSULFOKINE SIMULATOR (PSI) proteins from Arabidopsis (*Arabidopsis thaliana*), which are critical for plant growth^25^. Unlike Arabidopsis PSI1, Sr43 lacked a putative nuclear localization signal or a putative myristoylation site. Sr43 had no transmembrane domain, as predicted by InterPro. However, we established that Sr43 probably localizes to the nucleus, cytoplasm and plastids, as evidenced by the fluorescence detected from the transient expression of a *Sr43-GFP* (green fluorescent protein) construct in *Nicotiana benthamiana* leaf epidermal cells (Extended Data Fig. 3). The nuclear and cytoplasmic localization was confirmed in wheat protoplasts transfected with Sr43-GFP (Extended Data Fig. 3).

The domain structure of Sr43 was thus clearly different from that of proteins encoded by the ~290 cloned plant resistance genes, which were largely (73%) extracellular or intracellular immune receptors (Supplementary Table 1). To explore the unusual structure of Sr43 in more detail, we used the AlphaFold artificial intelligence–augmented system to generate a three-dimensional (3D) model^26^ (Supplementary Data 1). We determined that Sr43 adopts a modular structure, with the kinase and the two DUFs separated by flexible linker loops (Fig. 1e). The kinase domain contained α-helices and antiparallel β-strands, whereas the DUFs were entirely ⍺-helical. We compared the predicted structure of the Sr43 protein to those in the Protein Data Bank^27^. This identified structural similarities between DUF668 and some receptor-like protein kinase–like proteins outside of their kinase domains. We searched for ATP-binding sites using the small molecule docking program HADDOCK^28^ and identified one high-confidence ATP-binding site in DUF668 (Fig. 1f, Supplementary Table 11 and Supplementary Data 2).

**Fig. 2:**
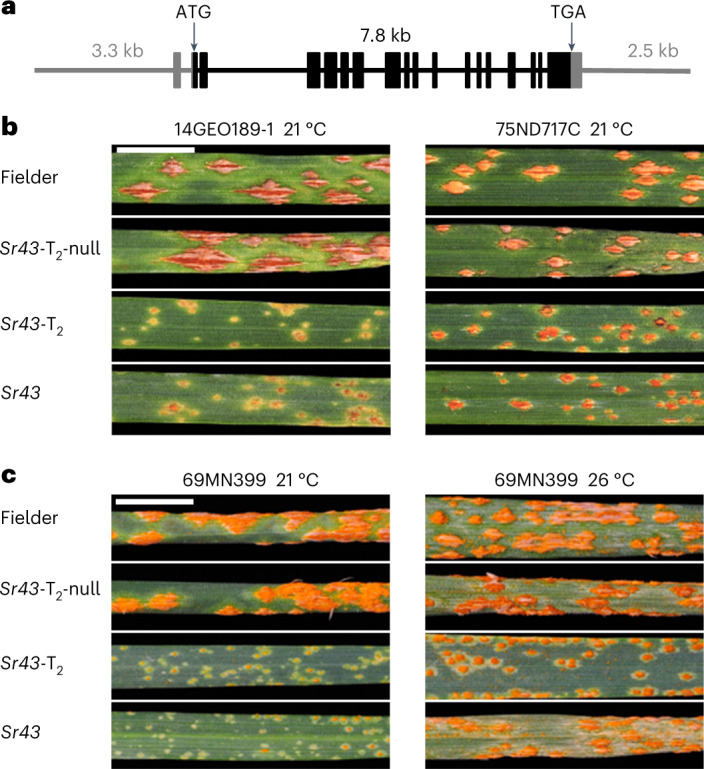
Confirmation of *Sr43* function, race specificity and temperature sensitivity. **a**, Schematic diagram of the *Sr43* genomic fragment used for transformation into wheat cv. Fielder. **b**, Representative leaves from seedlings of *Sr43* wild-type and transgenic lines alongside non-transgenic wild-type Fielder and null controls inoculated with *P*. *graminis* f. sp. *tritici* isolates 14GEO189-1 (avirulent on *Sr43*) and 75ND717C (intermediately virulent on *Sr43*). **c**, Effect of temperature on *Sr43*-mediated resistance to *Pgt* isolate 69MN399. Scale bar, 1 cm.

**Extended Data Fig. 4:**
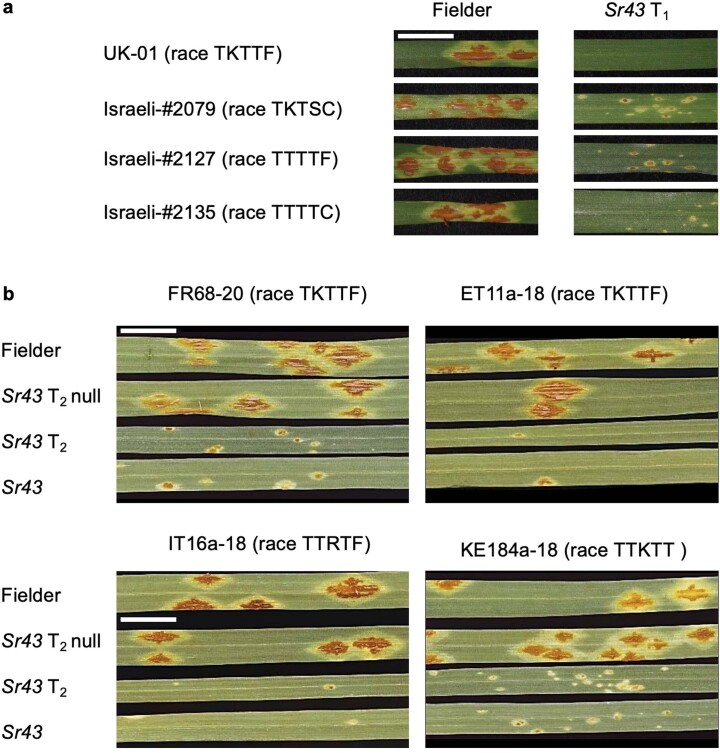
Reactions of Sr43 transgenic plants and controls to seven races/isolates of the fungal agent causing stem rust. **a**, homozygous transgenic lines from generation T_1_ alongside the non-transgenic parent cultivar Fielder. **b**, Homozygous transgenic lines and null segregants from generation T_2_. Scale bar, 1 cm.

We cloned a 14 kb genomic *Sr43* fragment including 3.2 kb of upstream and 2.5 kb of downstream regulatory sequence (Fig. 2a) (Supplementary Table 12) and introduced the resulting binary construct into the wheat cultivar Fielder. We obtained one primary (T_0_) transgenic plant and on the basis of quantitative PCR (qPCR) identified a genetically stable line with an estimated two copies of *Sr43* (Supplementary Tables 13–14 and Supplementary Fig. 17). We tested homozygous T_1_ and T_2_ lines against a geographically and phenotypically diverse panel of 11 *Pgt* isolates from North America, the Middle East, Europe and Africa. In ten cases, the *Sr43* transgenic and wild-type introgression lines were resistant, whereas the cultivars Chinese Spring (the introgression parent) and Fielder were susceptible (Fig. 2b, Extended Data Fig. 4a,b and Supplementary Table 15). By contrast, the *Pgt* isolate 75ND717C was intermediately virulent on the *Sr43* introgression and transgenic lines (Fig. 2b). For *Pgt* isolate 69MN399, we compared the phenotype at 21 °C and 26 °C and noticed a marked reduction in *Sr43*-mediated resistance at the higher temperature, in line with previous observations^29^ (Fig. 2c). Taken together, these results confirm (1) the wide-spectrum efficacy of *Sr43* (ref. ^29^), (2) that a 14 kb *Sr43* genomic fragment is sufficient for function and (3) that the transgenic line faithfully recapitulates the race-specific and temperature-sensitive resistance of wild-type *Sr43*.

**Fig. 3:**
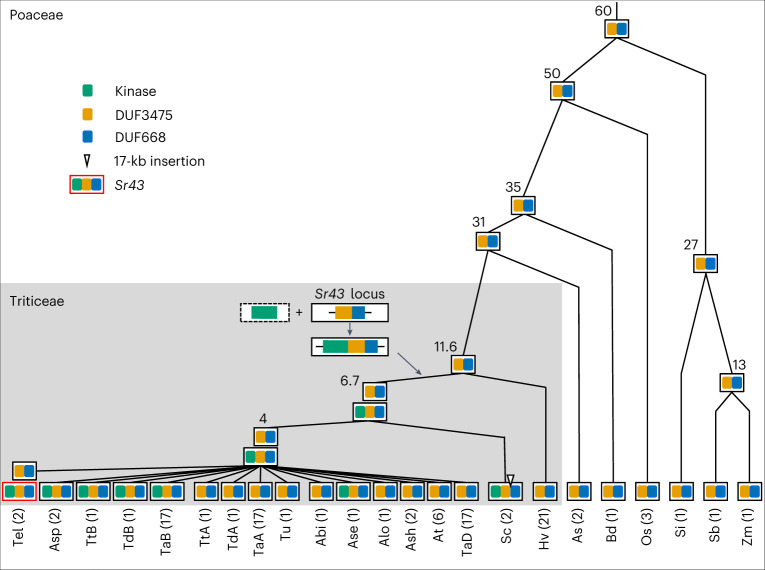
Phylogenetic tree of the Poaceae showing the origin and distribution of *Sr43* orthologs. The age of the last common ancestor in millions of years is indicated at each node on the basis of ref. ^23^. The Triticeae clade is highlighted in gray. Species are indicated at the bottom and abbreviated as follows: Tel, *Thinopyrum elongatum*; Asp, *Ae. speltoides*; TtB, *Triticum turgidum* ssp. *durum* B genome; TdB, *T. dicoccoides* B genome; TaB, *T. aestivum* B genome; TtA, *T. turgidum* ssp. *durum* A genome; TdA, *T. dicoccoides* A genome; TaA, *T. aestivum* A genome; Tu, *T. urartu*; Abi, *Aegilops bicornis*; Ase, *Ae. searsii*; Alo, *Ae. longissima*; Ash, *Aegilops sharonensis*; At, *Ae. tauschii*; TaD, *T. aestivum* D genome; Sc, *Secale cereale*; Hv, *Hordeum vulgare*; As, *Avena sativa*; Bd, *Brachypodium distachyon*; Os, *Oryza sativa*; Si, *Setaria italica*; Sb, *Sorghum bicolor*; Zm, *Zea mays*. The number of genomes analyzed for each species is indicated in parentheses.

**Extended Data Fig. 5:**
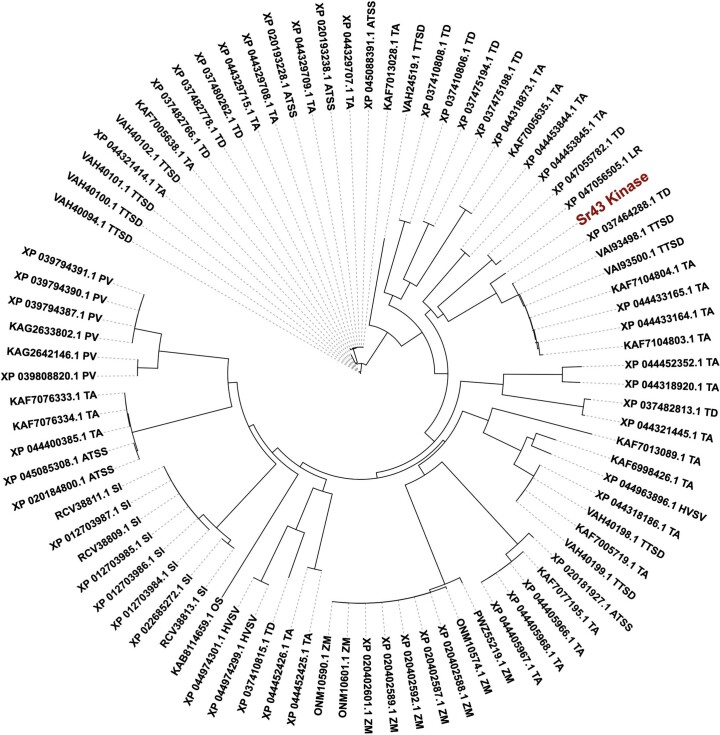
Phylogenetic tree of the Sr43 kinase domain with best hits from the NCBI protein database. A minimum threshold of 94% coverage and an identity of ≥60% were applied. ATSS, *Aegilops tauschii* subsp. *strangulata*; HVSV, *Hordeum vulgare* subsp. *vulgare*; LR, *Lolium rigidum*; OS, *Oryza sativa*; PV, *Panicum virgatum*; SI, *Setaria italica*; TA, *Triticum aestivum*; TD, *Triticum dicoccoides*; TTSD, *Triticum turgidum* subsp. *durum*; ZM, *Zea mays*.

**Extended Data Fig. 6:**
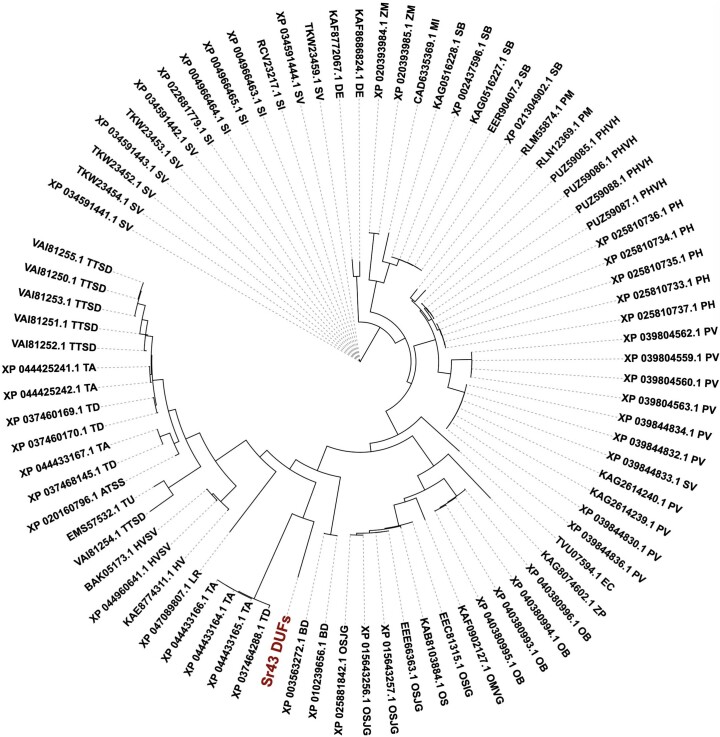
Phylogenetic tree of the Sr43 DUF region with best hits from the NCBI protein database. A minimum threshold of ≥99% coverage and an identity of ≥70% were applied. ATSS, *Aegilops tauschii* subsp. *strangulata*; BD, *Brachypodium distachyon*; DE, *Digitaria exilis*; EC, *Eragrostis curvula*; HV, *Hordeum vulgare*; HVSV, *Hordeum vulgare* subsp. *vulgare*; LR, *Lolium rigidum*; ML, *Miscanthus lutarioriparius*; OB, *Oryza brachyantha*; OMVG, *Oryza meyeriana* var. *granulata*; OS, *Oryza sativa*; OSIG, *Oryza sativa* Indica Group; OSJG, *Oryza sativa* Japonica Group; PH, *Panicum hallii*; PHVH, *Panicum hallii* var. *hallii*; PM, *Panicum miliaceum*; PV, *Panicum virgatum*; SI, *Setaria italica*; SV, *Setaria viridis*; SB, *Sorghum bicolor*; TA, *Triticum aestivu*m; TD, *Triticum dicoccoides*; TTSD, *Triticum turgidum* subsp. *durum*; TU, *Triticum urartu*; ZM, *Zea mays*; ZP, *Zizania palustris*.

We searched for Sr43 homologs to investigate its evolutionary origin. We identified proteins harboring either the kinase domain or the two DUFs alone across the Poaceae family spanning 60 million years of evolution (Supplementary Tables 16 and 17 and Extended Data Figs. 5 and 6). We detected the Sr43 protein domain arrangement only within the *Thinopyrum*, *Triticum*, *Aegilops* and *Secale* genera of the Triticeae tribe but not within *Hordeum*, suggesting that *Sr43* probably arose between 6.7 and 11.6 million years ago (Fig. 3 and Supplementary Table 18). In those lineages lacking a clear *Sr43* homolog, we mapped genes encoding the kinase and DUFs present in Sr43 to different chromosomes (for example, *Sorghum bicolor*, *Zea mays*, *T. urartu* and *Ae. sharonensis*) or on the same chromosome but 6–36 megabases (Mb) apart (*Ae. tauschii* and *Setaria italica*), suggesting that the recruitment of the kinase domain to the DUFs at the *Sr43* locus involved an ectopic recombination event (Supplementary Table 18). In *Thinopyrum elongatum*, the ancestral state and *Sr43* were retained as an intraspecies polymorphism; some species of *Aegilops* and *Triticum* retained the ancestral state (for example, *Ae. tauschii*), whereas others retained the *Sr43* innovation (for example, the *T. aestivum* and *T. durum* B genomes) (Fig. 3).

**Extended Data Fig. 7:**
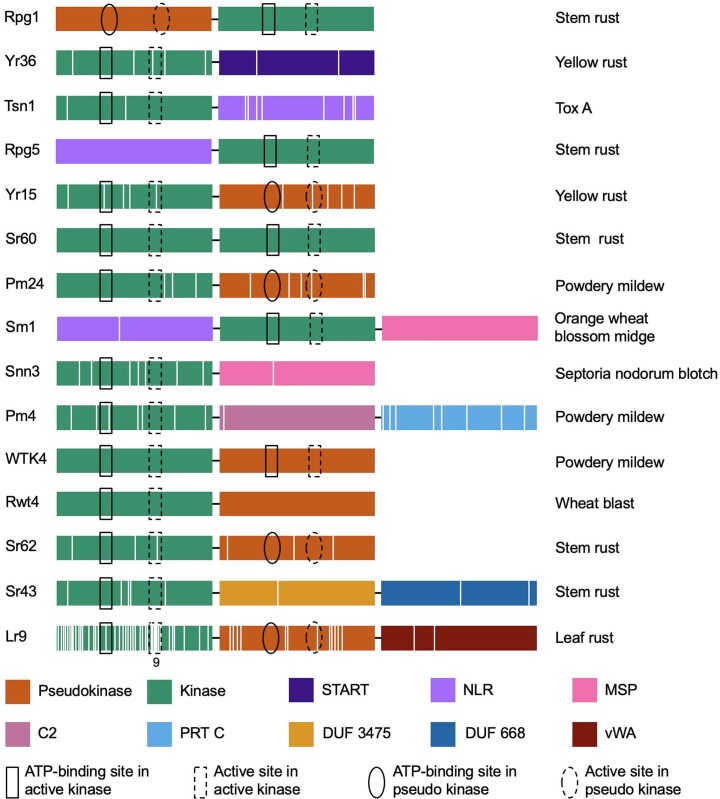
Schematic diagrams of Triticeae kinase fusion proteins with disease resistance function. The ATP-binding and active sites of the kinases are indicated by boxes and circles in the active and pseudo kinases, respectively, as determined by Interpro scanning, homology searching in AlphaFold-derived 3D models and/or BLAST. White bars indicate loss-of-function missense mutations obtained from EMS mutant screens. Eight mutations in the linker domains are not shown. The proteins are organized from top to bottom by date of publication: Rpg1, Yr36, Tsn1, Rpg5, Yr15, Sr60, Pm24, Sm1, Snn3, Pm4 (encoded by splice version 2), WTK4, Rwt4, Sr62 (refs. ^9–21^), Sr43 (this study) and Lr9 (ref. ^22^). START, Steroidogenic Acute Regulatory Protein-related Lipid Transfer; NLR, nucleotide binding and leucine-rich repeat; MSP, major sperm protein; C2, C2 domain; PRT C, phosphoribosyl transferase C-terminal domain; DUF, domain of unknown function; vWA, von Willebrand factor type A domain. White bars indicate the position of EMS-derived loss-of-function mutations (excluding early stop codon mutations) where they are available. The number 9 underneath the active site in the active kinase of Lr9 indicates the presence of nine mutations.

In summary, we cloned the wheat stem rust resistance gene *Sr43*, which encodes a protein kinase fused to two DUFs. Of the 82 Triticeae resistance genes cloned to date, most encode NLRs (*n* = 46), followed by protein kinase fusion proteins (*n* = 15) (Supplementary Table 19). Of the latter, seven are tandem kinases, whereas Sr43, Pm4, Snn3, Sm1, Tsn1, Yr36, Rpg5 and Lr9 are single or tandem protein kinases fused to different domains^16–22^ (Extended Data Fig. 7 and Supplementary Table 20). Little is known about the function of kinase fusion proteins but most confer race-specific resistance that is phenotypically indistinguishable from NLR-mediated resistance. Their encoding genes do not fall into the *Lr34*/*Lr67* category of adult, broad-spectrum and multipathogen resistance^30,31^. To explain the role of these kinases in resistance, we sought clues from NLRs whose modus operandi is now well understood. NLRs can act as guards that monitor host components targeted by pathogen effectors^32^. These guards detect the interaction between an effector and its target, leading to a conformational change in the NLR that triggers downstream defense responses. This tripartite interaction creates an evolutionary ‘tug-of-war’ that imposes selective pressure (1) on the effector to evade detection by the NLR while maintaining its ability to coerce the pathogenicity target, (2) on the NLR to recognize new effector variants and (3) on the pathogenicity target to avoid being disrupted by the effector while maintaining its cellular function. Duplication of the pathogenicity target can release it from this functional constraint and provide a ‘decoy’ for the effector. This diversification may also result in the decoy behaving genetically as the resistance gene^33^. In about 10% of all NLRs, the decoy has become integrated into the NLR itself^34^. Such a guardee–decoy fusion ensures that both components are inherited as a single operational unit.

**Extended Data Fig. 8:**
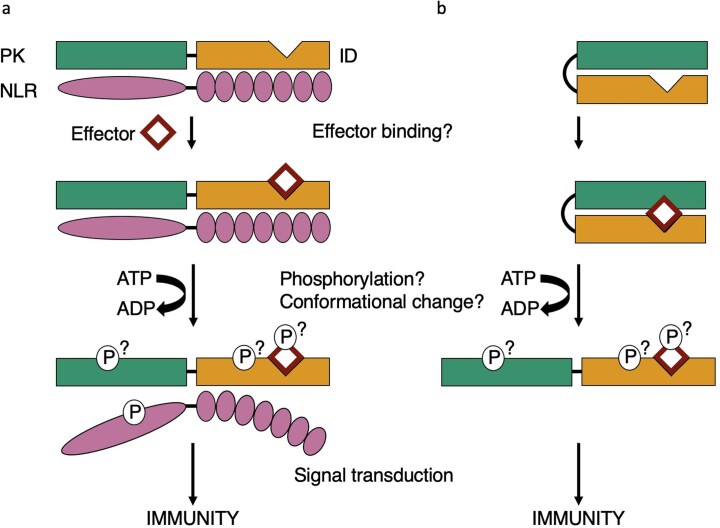
Mechanistic model of protein kinase fusion proteins in plant disease resistance. The protein kinase (PK) fusion protein contains an integrated decoy (ID) that traps the avirulence effector protein (red square). This triggers an autophosphorylation of either the protein kinase or decoy, the effector, or the nucleotide binding leucine-rich repeat (NLR) guard. This results in a conformational change which in turn triggers a signal cascade leading to downstream defense responses and immunity.

By extrapolation, protein kinase fusion proteins may be pathogenicity targets that are guarded by NLRs. All protein kinase fusion proteins have one apparent functional kinase that is fused to a second, typically non-functional, kinase domain but sometimes to an altogether different domain, as in for example Sr43, Lr9 and Pm4 (Extended Data Fig. 7). Perhaps as with those NLRs that carry an integrated decoy, this second domain might be an integrated decoy, while the apparent functional kinase exerts the signaling function. Indeed, plants produce various enzymes, including protein kinases with different integrated domains, to catalyze reactions of various substrates. In the case of protein kinase resistance proteins, the integrated domain would define the specific substrates of pathogen Avr proteins, whereas the kinase would catalyze the phosphorylation of the Avr protein, the integrated domain, itself, or a third signaling partner to trigger downstream defense, possibly via an NLR guard (Extended Data Fig. 8a). EMS mutagenesis of *Yr15*, *Pm24*, *Sr62* and *Sr43* has shown a preponderance of missense mutations affecting the kinase active site or ATP-binding pocket of the apparent functional kinase domain (Extended Data Fig. 7), supporting the notion that kinase-mediated signaling is required for function. Alternatively, Sr43 (and by extrapolation other kinase fusion resistance proteins)^35,36^ may function without an NLR cosignaling partner (Extended Data Fig. 8b).

The transgenic expression of *Sr43* in a different background allowed us to confirm the broad-spectrum efficacy of *Sr43*, highlighting its potential value in resistance breeding. However, it is possible to obtain gain-of-virulence pathogen mutants that have lost *AvrSr43* function under laboratory conditions^37^. Therefore, *Sr43* should be used in combination with other broad-spectrum resistance genes to maximize its longevity in the field.

## Methods

### Mutant collection development

We mutagenized 2,700 seeds of the wheat–*Th. elongatum* introgression line RWG34 containing *Sr43* (ref. ^29^). Dry seeds were incubated for 16 h with 200 ml of a 0.8% (w/v) EMS solution with constant shaking on a Roller Mixer (Model SRT1, Stuart Scientific) to ensure maximum homogenous exposure of the seeds to EMS. The excess solution was then removed and the seeds were washed three times with 400 ml of tap water. The M_1_ seeds were grown in the greenhouse and the seeds of M_2_ families (single heads) were collected. Eight seeds per M_2_ family were phenotyped with the *Pgt* isolate, race TPMKC. The M_3_ seeds derived from susceptible M_2_ plants were also tested to confirm that the M_2_ susceptible plants were true non-segregating mutants. To rule out seed contamination, ten mutants were verified using genotyping-by-sequencing (GBS)^38^. GBS data from the background (Chinese Spring), donor species (*Th. elongatum*, accession PI531737 from USDA-ARS GRIN), the Chinese Spring–*Th. elongatum Sr43* introgression line (RWG34) and the mutant lines were mapped to the reference genome sequence of Chinese Spring^39^ using BWA mem (v.0.7.12) with standard parameters^40^. Mapping results were sorted and converted to mpileup format using SAMtools^41^ (v.0.1.19). The mpileup files were examined with a previously published custom script linked to Zenodo^42^ to calculate the percentage of single-nucleotide polymorphisms from the donor that were shared with the introgression line per given interval. Several interval lengths were tested; a clear signal was observed for 10 Mb intervals.

### Chromosome flow sorting

Suspensions of mitotic metaphase chromosomes were prepared from root tips of the *Sr43* introgression line and eight EMS mutants as described by ref. ^43^ and ref. ^44^. Briefly, root tip meristem cells were synchronized using hydroxyurea, accumulated in metaphase using amiprophos-methyl and fixed in 2% (v/v) formaldehyde at 5 °C for 20 min. Intact chromosomes were released by mechanical homogenization of 100 root tips in 600 µl of ice-cold LB01 buffer^45^. GAA microsatellites were labeled on isolated chromosomes by fluorescence in situ hybridization in suspension (FISHIS) using 5′-FITC-GAA_7_-FITC-3′ oligonucleotides (Sigma) according to ref. ^46^ and chromosomal DNA was stained with 2 µg ml^–1^ of 4′,6-diamidino 2-phenylindole (DAPI). Chromosome analysis and sorting were conducted using a FACSAria II SORP flow cytometer and sorter (Becton Dickinson Immunocytometry Systems). Bivariate flow karyotypes plotting FITC and DAPI fluorescence were acquired for each sample and chromosomes were sorted at rates of 20–40 particles per second. Two batches of 55,000 and 70,000 copies of chromosome 7D with the *Th. elongatum* chromosome segment carrying *Sr43* were sorted from the wild-type *Sr43*-WT and one batch of 14,000–66,000 copies was sorted from the eight mutants into PCR tubes containing 40 μl of sterile deionized water (Supplementary Table 3).

Chromosome content of flow-sorted fractions was estimated by microscopy observations of 1,500–2,000 chromosomes sorted into a 10 μl drop of PRINS buffer containing 2.5% (w/v) sucrose^47^ on a microscope slide. Air-dried chromosomes were labeled by FISH with probes for the pSc119.2 repeat, Afa family repeat and 45S ribosomal DNA that allowed identification of all wheat and *Th. elongatum* chromosomes according to ref. ^48^. To determine chromosome contents and purity in the sorted fractions, at least 100 chromosomes in each flow-sorted sample were classified following the karyotype described by ref. ^49^.

### Sequencing and assembly of 7D–7E translocation chromosomes

The two separately flow-sorted chromosomal samples of the wild-type genotypes were used for preparation of two sequencing libraries. Chromosomal samples were treated with proteinase K (60 ng μl^−1^), after which DNA was purified without amplification. Chromosomal samples flow sorted from the mutants were treated similarly but their DNA contents were amplified to 2.5–12.6 μg by multiple displacement amplification (Supplementary Table 3) using an Illustra GenomiPhi v.2 DNA Amplification Kit (GE Healthcare) as described by ref. ^50^ and sequenced by Novogene. For the *Sr43* wild-type genotypes, 20 ng of non-amplified DNA was fragmented in a 20 μl volume using a Bioruptor Plus (Diagenode) five times for 30 s on the HIGH setting. Libraries for sequencing were prepared from fragmented DNA using an NEBNext Ultra II DNA Library Prep Kit for Illumina with the following modifications: (1) size selection was directed for larger final library size (~1,000 bp) and (2) PCR enrichment was done with six PCR cycles. Libraries were sequenced on a HiSeq2500 platform using a HiSeq Rapid SBS Kit v.2 as 250 bp paired-end reads. The raw data were trimmed for low-quality bases using Trimmomatic^51^ and assembled into scaffolds with Meraculous^52^ (v.2.0.5) using 111 nucleotide *k*-mers. Scaffolds shorter than 1 kb were eliminated. The assembly contained 168,523 scaffolds with a total assembly length of 1.29 gigabase (Gb). Among them, 25,581 scaffolds were longer than 13.9 kb with a total length 631.8 Mb.

### Candidate gene identification

Eight susceptible mutants derived from independent M_2_ families were selected for MutChromSeq mapping^53^. The raw reads from the eight mutants were individually mapped to the 10 kb chopped scaffold fragments using BWA^40^ (v.0.7.12) and SAMtools^41^ (v.1.8). One fragment was identified as having a single nucleotide mutation in all mutants. We calculated the probability of this being the candidate gene using formula number 4 developed by ref. ^12^, with 2,598 bp of *Sr43* coding sequence (CDS), assuming the average gene CDS is 1,000 bp in length and that chromosome 7D has 5,822 genes. All identified mutations were G-to-A or C-to-T transition mutations, which are typical of EMS mutagenesis.

### RNA extraction and *Sr43* annotation

Total RNA was extracted from the Chinese Spring–*Th. elongatum Sr43* introgression line with an RNeasy Plant Mini Kit (catalog no./ID 74904, Qiagen) following the manufacturer’s protocol and digested with Dnase I (Roche). RNA-seq was performed by Novogene. The RNA-seq reads were trimmed with Trimmomatic (http://www.usadellab.org/cms/?page=trimmomatic). Hisat2 (v.2.1.0)^54^ was used to map the short reads onto the *Sr43* genomic sequence. The SAM output file was converted into a BAM file using SAMtools^41^ (v.1.8) (http://www.htslib.org/) and sorted according to their position along the *Sr43* genomic sequence and indexed for visualization by IGV (https://software.broadinstitute.org/software/igv/). To determine the alternative splicing of *Sr43*, we constructed a full-length cDNA library using a SMARTer PCR cDNA Synthesis kit (catalog no. 634926, Clontech/TaKaRa). Transcripts corresponding to each of the four splice variants were identified by Sanger sequencing of 20 clones obtained from transformation of long-range PCR on the full-length cDNA library with primers specific to the *Sr43* 5′ and 3′ ends (Supplementary Table 5).

### Engineering of the *Sr43* binary vector construct

On the basis of the gene annotation, three overlapping segments of the *Sr43* gene were PCR-amplified (Supplementary Table 12) with high-fidelity Q5 DNA polymerase (NEB) following the manufacturer’s instructions. The PCR products were purified with a QIAquick PCR Purification kit (QIAGEN) and A-tailed using *Taq* DNA polymerase before being cloned into the pCR2.1 vector (TOPO PCR Cloning Kits-K202020, Thermo Fisher Scientific). The positive clones were digested with three sets of restriction enzymes, NotI, NotI-PvuI and PvuI-PmeI (NEB), to generate *Sr43* fragment parts 1, 2 and 3, respectively. The digested fragments were gel-purified and then parts 2 and 3 were combined in a three-way ligation reaction with the binary vector pGGG-AH-NotI/PmeI^12^ digested with NotI and PmeI, using T4 DNA ligase (M0202S, NEB). Subsequently, the binary construct was linearized with NotI and part 1 was dropped in. A positive clone with part 1 in the correct orientation, pGGG-*Sr43*, was verified by Sanger sequencing. The pGGG-*Sr43* is available from Addgene under accession number 186974.

### Wheat transformation

The binary construct pGGG-*Sr43* was transformed into wheat cv. Fielder using *Agrobacterium tumefaciens*-mediated transformation^55^. The *Sr43* copy number was postulated by testing the copy number of the *hygromycin B phosphotransferase* selectable marker in T_0_ and T_1_ plants by iDNA Genetics using qPCR^56^. From a non-segregating genetically stable T_1_ family we advanced a T_2_ line (BW_30183) for further copy number testing. We designed gene-specific primers for *Sr43*, the *hygromycin B phosphotransferase* selectable marker gene and single-copy, three-copy and six-copy wheat endogenous control genes (Supplementary Table 13). The primer sequences for the endogenous genes were designed on the basis of the cv. Fielder reference genome^57^. DNA was extracted from a single T_3_ plant (derived from the T_2_ family BW_30183) using the Qiagen genomic DNA extraction kit (Qiagen, catalog no. 19060) with 500 per g columns (Qiagen, lot 169047970) following the QIAGEN Genomic DNA Handbook. The qPCR was done in a 10 μl reaction with 1X SsoAdvanced Universal SYBR Green Supermix (BioRad), 0.5 μM primer and 2 ng μl^−1^ of DNA using an initial denaturation at 95 °C for 3 min, followed by a denaturation at 95 °C for 15 s and annealing + extension at 60 °C for 30 s, for 40 cycles on a CFX96 Real-Time PCR system. The *Sr43* gene copy number was calculated on the basis of the endogenous reference genes (Supplementary Fig. 17 and Supplementary Table 14).

### Stem rust phenotyping

The stem rust tests were carried out in a greenhouse or in growth chambers. The greenhouse/growth chambers were maintained at 21 °C with a 14 h light period and ~40% relative humidity. Plants were inoculated with *P. graminis* f. sp. *tritici* when the second leaf was fully expanded, 10–12 days after sowing, at a rate of ~0.12 mg of spores per plant. After a 16 h incubation period in the dark under high humidity (100%) conditions, inoculated seedlings were returned to the greenhouse/growth chamber and then scored for reaction to stem rust 12–14 days later. The infection types were recorded using the Stakman scale^58^. For temperature sensitivity tests, the high temperature was set to 26 °C. The *Pgt* races used in this study were TPMKC (isolate 74MN1409) from the United States; QTHJC (isolates 75ND717C and 69MN399) from the United States; TKTTF (isolate ET11a-18) from Ethiopia; TTKTT (isolate KE184a/18) from Kenya; TKTSC (isolate IS no. 2079), TTTTF (isolate IS no. 2127) and TTTTC (isolate IS no. 2135) from Israel; TKTTF (isolate FR68-20) from France; TTRTF (isolate IT16a-18) from Italy; TKTTF (isolate UK-01) from the United Kingdom; and TRTTF (isolate 14GEO189-1) from Georgia (Supplementary Table 15).

### Protein homology searches

We used InterPro v.88.0 to search for protein family domains in Sr43, for example, a transmembrane domain^59^. To check for the presence of myristoylation sites and nuclear localization signals, we used Myristoylator^60^ and cNLS mapper^61^, respectively (accessed 11 March 2023).

### Sr43 protein 3D modeling and ATP-binding site prediction

We used the open source code of AlphaFold v.2.0 (ref. ^26^) and the supercomputer of King Abdullah University of Science and Technology, Shaheen II (https://www.hpc.kaust.edu.sa/) through the multinode system Ibex (https://www.hpc.kaust.edu.sa/ibex). We input the amino acid sequence of Sr43 and the output was five unrelaxed, five relaxed and five ranked models in .pdb format. We used the ranked_1.pdb model that contains the predictions with the highest confidence with the best local distance difference test (lDDT) score standing at 70.76. We next input the ranked_1.pdb model obtained from Alphafold and each domain separately into the protein structure comparison server Dali^27^.

We used HADDOCK2.4, a web server for small molecule binding site screening^28^, to screen the DUF2 domain for potential ATP-binding sites. The input files consisted of the DUF2 domain of Sr43 after removing all loops from the .pdb file and ATP in .pdb format. The settings used were:

Define randomly ambiguous interaction restraints from accessible residues—ON

Number of structures for rigid body docking—10,000

Number of structures for semiflexible refinement—400

Number of structures for the final refinement—400

Clustering method (RMSD or fraction of common contacts (FCC))—RMSD

RMSD cutoff for clustering (recommended: 7.5 A for RMSD, 0.60 for FCC)—2.0

Evdw 1—1.0

Eelec 3—0.1

Initial temperature for second TAD cooling step with flexible side-chain at the interface—500

Initial temperature for third TAD cooling step with fully flexible interface—300

Number of MD steps for rigid body high temperature TAD—0

Number of MD steps during first rigid body cooling stage—0

The output files were ten clusters of different predicted ATP-binding sites. The cluster with the best prediction score (*Z*-score) was cluster 6.

### Expression and purification of recombinant Sr43 protein

The native CDS of *Sr43* plus two additional nucleotides (CC) at the beginning of the CDS (to maintain the open reading frame with His_6_ tag) was commercially synthesized (Twist Bioscience) and cloned into the Gateway entry vector pTwist_ENTR. For recombinant protein expression, *Sr43* was transferred into the expression vector pDEST-His_6_-MBP by Gateway LR clonase reaction (Invitrogen). The resulting clone was verified by Sanger sequencing.

His_6_-MBP-Sr43 tagged protein was expressed in the *E. coli* Rosetta strain by growing the bacterial culture to an optical density OD_600_ of 0.8 at 37 °C and then inducing the protein expression by addition of 0.5 mM isopropyl β-d-thiogalactopyranoside at 18 °C and further incubating the culture for 14–16 h. The recombinant protein was purified under native conditions using Ni-NTA agarose beads (Invitrogen catalog no. R901-15) following the manufacturer’s instructions.

### In vitro kinase reaction and phosphosite identification

The buffer composition of the purified His_6_-MBP-Sr43 protein was changed to the kinase reaction buffer (20 mM Tris-HCl pH 7.5, 10 mM MgCl_2_, 5 mM EGTA, 1 mM DTT and 50 μM ATP) using PD10 desalting columns (GE Healthcare). His_6_-MBP-Sr43 was mixed with a commercial substrate maltose-binding protein DNA gyrase (Prospec Protein Specialists PRO-616) and incubated at ambient temperature for 30 min. After adding SDS-sample buffer to stop the reaction, the protein was denatured by boiling at 95 °C for 10 min. SDS–polyacrylamide gel electrophoresis was used to resolve protein samples. The gel was stained with SimplyBlue SafeStain (Novex cat. no. LC6065) and the band that corresponded to the protein of interest was excised, cut into pieces of 0.5 mm^3^ and destained with four sequential washes of 15 min each with acetonitrile and 100 mM NH_4_HCO_3_. The proteins in the gel pieces were reduced with 10 mM Tris (2-carboxyethyl) phosphine hydrochloride (TCEP, C-4706 Sigma) in 100 mM NH_4_HCO_3_ at 37 °C for 1 h. Then the reduced disulfide bonds were alkylated with 50 mM iodoacetamide at ambient temperature for 30 min. Following reduction and alkylation of proteins, they were digested with trypsin (porcine trypsin, Promega) at 37 °C overnight. Formic acid was added to a final concentration of 1% to stop the digestion and the tryptic peptides were recovered by incubating the gel pieces in acetonitrile. The recovered peptides were desalted using Sep-Pak C18 1 ml vac cartridge (Waters SKU: WAT023590) and analyzed by liquid chromatography with tandem mass spectrometry (LC-MS/MS) (Supplementary Fig. 14). Peptide samples were separated using a C18 column linked to an Orbitrap Fusion Lumos mass spectrometer (Acclaim PepMap C18, 25 cm length 75 m I.D. 3 m particle size, 100 porosity, Dionex). The LC gradient increased from 5% solvent B (water/ACN/formic acid, 20/80/0.1, v/v/v) to 45% solvent B over 45 min, then to 90% solvent B for 10 min. Using HCD fragmentation in the Orbitrap Fusion Lumos instrument, the MS instrument recorded fragmentation spectra on the top ten peptides. Using the msConvert interface, the RAW data files were converted to MGF files. The Mascot server was used to conduct database searches and the following criteria were used: (1) database containing the amino acid sequences of Sr43, MBP and contaminant proteins; (2) enzymatic specificity (trypsin permitting two allowed missed cleavages); (3) cysteine residues are fixedly modified (carbamidomethyl); (4) phosphorylation of S, T and Y residues may be variably modified; (5) precursor masses are tolerable to 5 ppm; (5) fragment ions are tolerable to 0.02 Da. Mascot and MD scores were used to filter the findings. The peptides identified for determining the protein coverage of maltose-binding protein are shown when incubated alone and when incubated with His6-MBP-Sr43 (Supplementary Figs. 15 and 16).

### Sr43 protein localization in *N. benthamiana*

To generate the 35S:*Sr43-GFP* construct, a codon-optimized open reading frame of *Sr43* splice version 1 was synthesized and ligated into pDONR221 (Twist Bioscience). The entry clone was then introduced into the binary vector pB7FGW2,0 by single Gateway LR reaction (Invitrogen). The 35S:*PLSP2A-mRFP* construct was generated by combining an entry clone of the *PLSP2A* CDS with the *p35S* promoter and the *mRFP* fluorescent reporter using sequential Gateway cloning.

The 35S:*Sr43-GFP* and 35S:*PLSP2A-mRFP* constructs were transferred into *A. tumefaciens* strain GV3101 and infiltrated into tobacco leaves as described in ref. ^62^. The GFP signal was excited at 488 nm and detected between 500 and 535 nm. The mRFP signal was excited at 555 nm and detected between 566 and 646 nm. Images were acquired using an inverted Leica SP8 Stellaris FALCON with an HC PL APO 63× 1,2 W CORR UVIS CS2 objective.

### Sr43 protein localization in wheat protoplasts

To generate the pZmUbi:*GFP-Sr43* construct, the splice version 1 *Sr43* CDS was subcloned from the pDONR221 mentioned above into the pJET1.2 vector (Thermo Fisher) as a level I module for further Golden Gate cloning^63^. The level II expression construct was assembled with the level II backbone (BB10), the level I ZmUbiquitin promoter, the level I *GFP*-tag, the level I *Sr43* and the level I NOS terminator via a BsaI cut-ligation reaction^63^. The pZmUbi:*NLS-mCherry* construct was generated in the same way by combining the level II backbone, the level I ZmUbqiuitin promoter, the level I nuclear localization sequence, the level I *mCherry* and the level I NOS terminator. The pZmUbi:*GFP* control was generated by transferring the GFP CDS via LR clonase reaction to pZmUbi:GW^64^.

Plasmid DNAs were purified from *E. coli* harboring pZmUbi:*GFP-Sr43*, pZmUbi:*NLS-mCherry*, pZmUbi:*GFP* with NucleoBond Xtra Maxi Plus Kit (Macherey-Nagel). Mesophyll cells were isolated from 9-day-old wheat seedlings (cv. Fielder) grown in short-day conditions (8 h light, 16 h dark). Protoplast isolation and transfection were performed as described in ref. ^64^.

Florescence was observed with a Carl Zeiss LSM upright 880 Axio Imager 2 confocal microscope with a Plan-Apochromat 63×/1.4 Oil DIC M27 objective. GFP was excited using an argon laser (488 nm) and detected between 494 and 552 nm. The mCherry was excited using a Diode Pumped Solid State laser (561 nm) and florescence was detected between 596 and 649 nm.

### Phylogenetic analysis

We constructed a phylogenetic tree on the basis of the aligned protein sequences of 100 best hits (ID ≥75%) of the kinase domain sequence and Sr43 DUF region sequence against the NCBI protein database. The phylogenetic tree (neighbor-joining method) for kinase and DUF domains were computed with Clustal Omega (https://www.ebi.ac.uk/Tools/msa/clustalo/) and drawn with iTOL (https://itol.embl.de/).

### Reporting summary

Further information on research design is available in the Nature Portfolio Reporting Summary linked to this article.

## Online content

Any methods, additional references, Nature Portfolio reporting summaries, source data, extended data, supplementary information, acknowledgements, peer review information; details of author contributions and competing interests; and statements of data and code availability are available at 10.1038/s41588-023-01402-1.

## Supplementary information

Supplementary Information Supplementary Figs. 1–17.

Reporting Summary

Peer Review File

Supplementary Tables Supplementary Tables 1–20

Supplementary Data 1 Three-dimensional model of Sr43, as predicted by AlphaFold.

Supplementary Data 2 Three-dimensional model of Sr43 with ATP, as predicted by AlphaFold.

## Data Availability

The datasets generated during and/or analyzed in the current study are publicly available as follows. The sequence reads were deposited in the European Nucleotide Archive under project numbers PRJEB52878 (GBS data), PRJEB51958 (chromosome flow-sorted data) and PRJEB52088 (RNA-seq data). The *Sr43* gene and transcript sequence were deposited in NCBI Genbank under accession number ON237711. The *Sr43* chromosome assembly has been deposited in Zenodo (10.5281/zenodo.6777941). The following public databases/datasets were used in the study: Chinese Spring reference genome^39^, Gramene (http://www.gramene.org/), https://ensembl.gramene.org/Multi/Tools/Blast, https://wheat.pw.usda.gov/GG3/blast, BLAST non-redundant protein sequence (https://blast.ncbi.nlm.nih.gov/Blast.cgi?PROGRAM=blastx&PAGE_TYPE=BlastSearch&LINK_LOC=blasthome), Taxonomy Browser (https://www.ncbi.nlm.nih.gov/Taxonomy/Browser/wwwtax.cgi?id=1437183), AlphaFold^26^ (https://alphafold.ebi.ac.uk), Dali^27^ (http://ekhidna2.biocenter.helsinki.fi/dali/) and HADDOCK^28^ (https://www.bonvinlab.org/education/HADDOCK-binding-sites/.

## Code availability

The scripts used in these analyses have been published in GitHub (https://github.com/steuernb/GBS_introgression_line_analysis) and linked with Zenodo (https://zenodo.org/badge/latestdoi/394326594)^42^.

## References

[1] Hafeez AN (2021). Creation and judicious application of a wheat resistance gene atlas. Mol. Plant.

[2] Knott DR (1977). Transfer to wheat and homoeology of an *Agropyron elongatum* chromosome carrying resistance to stem rust. Can. J. Genet. Cytol..

[3] Kibiridge-Sebunya I, Knott DR (1983). Transfer of stem rust resistance to wheat from an *Agropyron* chromosome having a gametocidal effect. Can. J. Genet. Cytol..

[4] Savary S (2019). The global burden of pathogens and pests on major food crops. Nat. Ecol. Evol..

[5] van Esse P (2020). Genetic modification to improve disease resistance in crops. New Phytol..

[6] McDonald BA, Linde C (2002). Pathogen population genetics, evolutionary potential and durable resistance. Annu. Rev. Phytopathol..

[7] Luo M (2021). A five-transgene cassette confers broad-spectrum resistance to a fungal rust pathogen in wheat. Nat. Biotechnol..

[8] Kourelis J, van der Hoorn RAL (2018). Defended to the nines: 25 years of resistance gene cloning identifies nine mechanisms for R protein function. Plant Cell.

[9] Brueggeman R (2002). The barley stem rust-resistance gene *Rpg1* is a novel disease-resistance gene with homology to receptor kinases. Proc. Natl Acad. Sci. USA.

[10] Klymiuk V (2018). Cloning of the wheat *Yr15* resistance gene sheds light on the plant tandem kinase-pseudokinase family. Nat. Commun..

[11] Chen S (2020). Wheat gene *Sr60* encodes a protein with two putative kinase domains that confers resistance to stem rust. New Phytol..

[12] Yu G (2022). *Aegilops sharonensis* genome-assisted identification of stem rust resistance gene *Sr62*. Nat. Commun..

[13] Lu P (2020). A rare gain of function mutation in a wheat tandem kinase confers resistance to powdery mildew. Nat. Commun..

[14] Gaurav K (2022). Population genomic analysis of *Aegilops tauschiii* identifies targets for bread wheat improvement. Nat. Biotechnol..

[15] Arora S (2023). A wheat kinase and immune receptor form host-specificity barriers against the blast fungus. Nat. Plants.

[16] Fu D (2009). A Kinase-START gene confers temperature-dependent resistance to wheat stripe rust. Science.

[17] Sánchez-Martín J (2021). Wheat *Pm4* resistance to powdery mildew is controlled by alternative splice variants encoding chimeric proteins. Nat. Plants.

[18] Zhang Z (2021). A protein kinase–major sperm protein gene hijacked by a necrotrophic fungal pathogen triggers disease susceptibility in wheat. Plant J..

[19] Faris JD (2010). A unique wheat disease resistance-like gene governs effector-triggered susceptibility to necrotrophic pathogens. Proc. Natl Acad. Sci. USA.

[20] Arora D (2013). Allele characterization of genes required for *rpg4*-mediated wheat stem rust resistance identifies *Rpg5* as the *R* gene. Phytopathology.

[21] Walkowiak S (2020). Multiple wheat genomes reveal global variation in modern breeding. Nature.

[22] Wang, Y. et al. An unusual tandem kinase fusion protein confers leaf rust resistance in wheat. (2022); 10.1038/s41588-023-01401-2 10.1038/s41588-023-01401-2 PMC10260399 37217716

[23] Chalupska D (2008). *Acc* homoeoloci and the evolution of wheat genomes. Proc. Natl Acad. Sci. USA.

[24] International Wheat Genome Sequencing Consortium (IWGSC (2014). A chromosome-based draft sequence of the hexaploid bread wheat (*Triticum aestivum*) genome. Science.

[25] Stührwohldt N (2014). The PSI family of nuclear proteins is required for growth in *Arabidopsis*. Plant Mol. Biol..

[26] Jumper J (2021). Highly accurate protein structure prediction with AlphaFold. Nature.

[27] Holm L, Rosenström P (2010). Dali server: conservation mapping in 3D. Nucleic Acids Res..

[28] van Zundert GCP (2016). The HADDOCK2.2 web server: user-friendly integrative modelling of biomolecular complexes. J. Mol. Biol..

[29] Niu Z (2014). Development and characterization of wheat lines carrying stem rust resistance gene *Sr43* derived from *Thinopyrum ponticum*. Theor. Appl. Genet..

[30] Krattinger SG (2009). A putative ABC transporter confers durable resistance to multiple fungal pathogens in wheat. Science.

[31] Moore JW (2015). A recently evolved hexose transporter variant confers resistance to multiple pathogens in wheat. Nat. Genet..

[32] Jones J, Dangl J (2006). The plant immune system. Nature.

[33] van der Hoorn RA, Kamoun S (2008). From guard to decoy: a new model for perception of plant pathogen effectors. Plant Cell.

[34] Cesari S (2014). A novel conserved mechanism for plant NLR protein pairs: the ‘integrated decoy’ hypothesis. Front. Plant Sci..

[35] Sánchez-Martín J, Keller B (2021). NLR immune receptors and diverse types of non-NLR proteins control race-specific resistance in Triticeae. Curr. Opin. Plant Biol..

[36] Klymiuk V (2021). Tandem protein kinases emerge as new regulators of plant immunity. Mol. Plant–Microbe Interact..

[37] Kangara N (2020). Mutagenesis of *Puccinia graminis* f. sp. *tritici* and selection of gain-of-virulence mutants. Front. Plant Sci..

[38] Poland JA, Rife TW (2012). Genotyping-by-sequencing for plant breeding and genetics. Plant Genome.

[39] International Wheat Genome Sequencing Consortium (IWGSC (2018). Shifting the limits in wheat research and breeding using a fully annotated reference genome. Science.

[40] Li H, Durbin R (2009). Fast and accurate short read alignment with Burrows–Wheeler transform. Bioinformatics.

[41] Li H (2009). 1000 Genome Project Data Processing Subgroup, The Sequence alignment/map (SAM) format and SAMtools. Bioinformatics.

[42] Yu, G. et al. Reference genome-assisted identification of stem rust resistance gene *Sr62* encoding a tandem kinase. *Nat. Commun.* **13**, 1607 (2022). 10.1038/s41467-022-29132-8 PMC8956640 35338132

[43] Vrána J (2000). Flow sorting of mitotic chromosomes in common wheat (*Triticum aestivum* L.). Genetics.

[44] Kubaláková M (2002). Flow karyotyping and chromosome sorting in bread wheat (*Triticum aestivum* L.). Theor. Appl. Genet..

[45] Doležel J (1989). Analysis of nuclear DNA content in plant cells by flow cytometry. Biol. Plant..

[46] Giorgi D (2013). FISHIS: fluorescence *in situ* hybridization in suspension and chromosome flow sorting made easy. PLoS ONE.

[47] Kubaláková M (1997). Mapping of repeated DNA sequences in plant chromosomes by PRINS and C-PRINS. Theor. Appl. Genet..

[48] Molnár I (2016). Dissecting the U, M, S and C genomes of wild relatives of bread wheat (*Aegilops* spp.) into chromosomes and exploring their synteny with wheat. Plant J..

[49] Gaál E (2018). Identification of COS markers specific for *Thinopyrum elongatum* chromosomes preliminary revealed high level of macrosyntenic relationship between the wheat and *Th. elongatum* genomes. PLoS ONE.

[50] Šimková H (2008). Coupling amplified DNA from flow-sorted chromosomes to high-density SNP mapping in barley. BMC Genomics.

[51] Bolger AM (2014). Trimmomatic: a flexible trimmer for Illumina sequence data. Bioinformatics.

[52] Chapman JA (2015). A whole-genome shotgun approach for assembling and anchoring the hexaploid bread wheat genome. Genome Biol..

[53] Sánchez-Martín J (2016). Rapid gene isolation in barley and wheat by mutant chromosome sequencing. Genome Biol..

[54] Kim D (2019). Graph-based genome alignment and genotyping with HISAT2 and HISAT-genotype. Nat. Biotechnol..

[55] Hayta S (2019). An efficient and reproducible *Agrobacterium*-mediated transformation method for hexaploid wheat (*Triticum aestivum* L.). Plant Methods.

[56] Bartlett JG (2008). High-throughput *Agrobacterium*-mediated barley transformation. Plant Methods.

[57] Sato K (2021). Chromosome-scale genome assembly of the transformation-amenable common wheat cultivar ‘Fielder’. DNA Res..

[58] Stakman E. C. et al. *Identification of Physiologic Races of* Puccinia graminis *var.* tritici (USDA, 1962).

[59] Blum M (2020). The InterPro protein families and domains database: 20 years on. Nucleic Acids Res..

[60] Bologna G (2004). N-terminal myristoylation predictions by ensembles of neural networks. Proteomics.

[61] Kosugi S (2009). Systematic identification of yeast cell cycle-dependent nucleocytoplasmic shuttling proteins by prediction of composite motifs. Proc. Natl Acad. Sci. USA.

[62] Aljedaani F (2021). A semi-in vivo transcriptional assay to dissect plant defense regulatory modules. Methods Mol. Biol..

[63] Binder A (2014). A modular plasmid assembly kit for multigene expression, gene silencing and silencing rescue in plants. PLoS ONE.

[64] Saur IML (2019). A cell death assay in barley and wheat protoplasts for identification and validation of matching pathogen AVR effector and plant NLR immune receptors. Plant Methods.

