# Supplementary material for: The wheat stem rust resistance gene Sr43 encodes an unusual protein kinase
Source: Nat Genet. 2023 May 22;55(6):921–6. doi: 10.1038/s41588-023-01402-1 (PMC10260397; doi:10.1038/s41588-023-01402-1)
Supplement: Supplementary file 1 — Supplementary Figs. 1–17. [file 41588_2023_1402_MOESM1_ESM.pdf]

nature genetics

Letter

https://doi.org/10.1038/s41588-023-01402-1

The wheat stem rust resistance gene *Sr43* encodes an unusual protein kinase

In the format provided by the authors and unedited

Table of Contents

Supplementary Fig. 1: Genotyping-by-sequencing (GBS) plot of the *Sr43* wheat-*Th. elongatum* introgression wild type line……………………………………………..2

Supplementary Fig. 2: GBS plot of the *Sr43* wheat-*Th. elongatum* introgression EMS mutant 31b……………………………………………..………………………..3

Supplementary Fig. 3: GBS plot of the *Sr43* wheat-*Th. elongatum* introgression EMS mutant 187g. ……………………………………..………………………..4

Supplementary Fig. 4: GBS plot of the *Sr43* wheat-*Th. elongatum* introgression EMS mutant 276b. …………………………………………..……………………..5

Supplementary Fig. 5: GBS plot of the *Sr43* wheat-*Th. elongatum* introgression EMS mutant 450b. ………………………………………………..…………………..6

Supplementary Fig. 6: GBS plot of the *Sr43* wheat-*Th. elongatum* introgression EMS mutant 706f……………………….…………………………………………..7

Supplementary Fig. 7: GBS plot of the *Sr43* wheat-*Th. elongatum* introgression EMS mutant 1013a……………………….………………………………………..8

Supplementary Fig. 8: GBS plot of the *Sr43* wheat-*Th. elongatum* introgression EMS mutant 1102h……………………….………………………………………..9

Supplementary Fig. 9: GBS plot of the *Sr43* wheat-*Th. elongatum* introgression EMS mutant 1298g……………………….……………………………………10

Supplementary Fig. 10: GBS plot of the *Sr43* wheat-*Th. elongatum* introgression EMS mutant 1513d…………………………………………………………11

Supplementary Fig. 11: GBS plot of the *Sr43* wheat-*Th. elongatum* introgression EMS mutant 1671a…………………………………………………………12

Supplementary Fig. 12: Amino acid alignment between Sr43 and STKc IRAK..….13

Supplementary Fig. 13: Amino acid alignment between Sr43 and cAPK-α……..….14

Supplementary Fig. 14. Schematic representation of the phosphorylation reaction and MASCOT search results…….……………………..……………………15

Supplementary Fig. 15. Mass spectra of the peptides that are targeted by His6-MBP-Sr43 for phosphorylation of residues T54, S74, S115, T129 and Y177..…….…….16

Supplementary Fig. 16. Mass spectra of the peptides that are targeted by His6-MBP-Sr43 for phosphorylation of residues S234, T250, S256, S264 and S307…..…..….17

Supplementary Fig. 17, Regression of calculated reference gene copy number on theoretical reference gene copy number……………………..………………...18

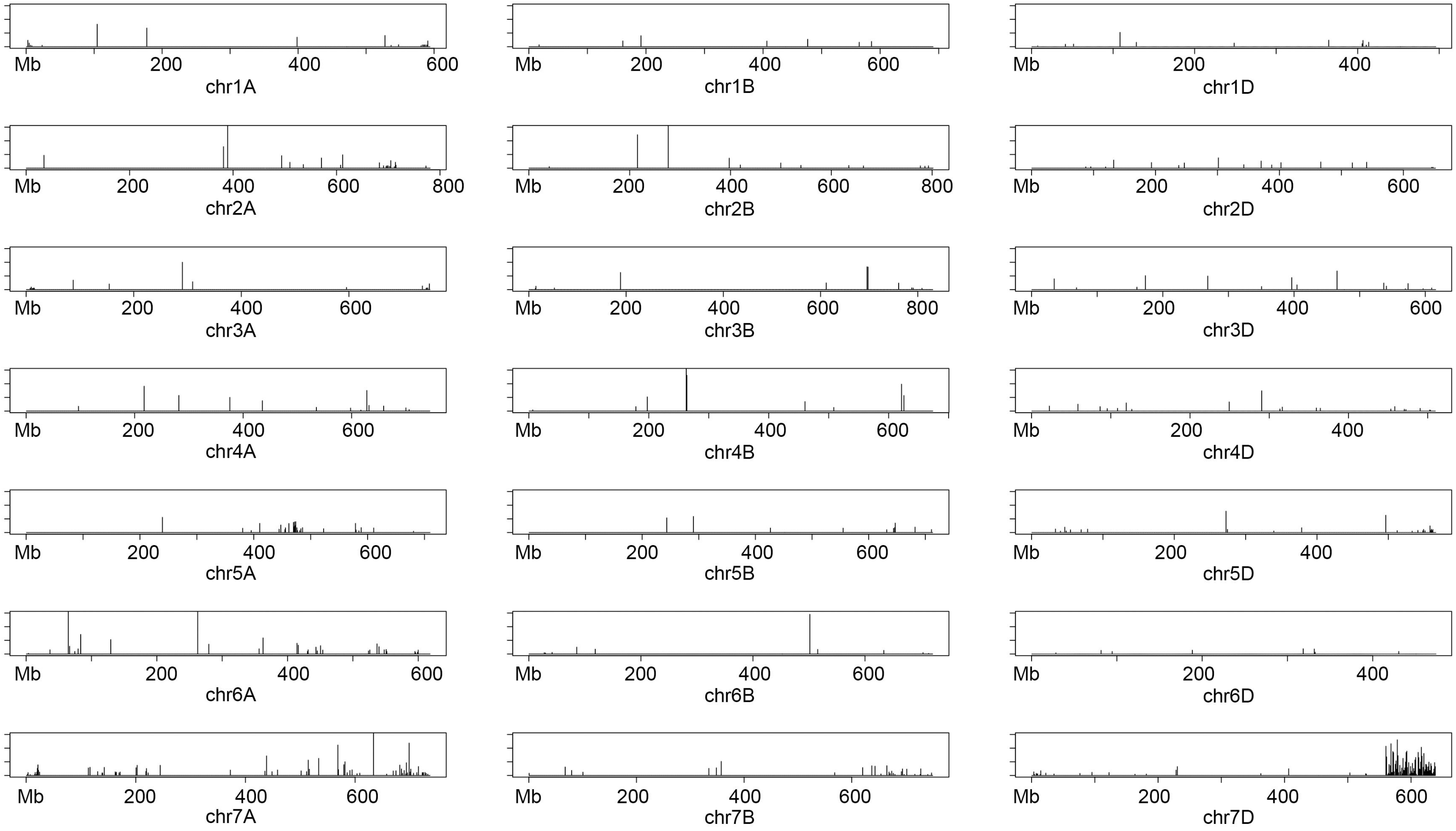

Supplementary Fig. 1: Genotyping-by-sequencing (GBS) plot of the *Sr43* wheat-*Th. elongatum* introgression wild type line. The SNP frequency was plotted in 10 Mb bins along the length of each chromosome relative to wheat cv. Chinese Spring.

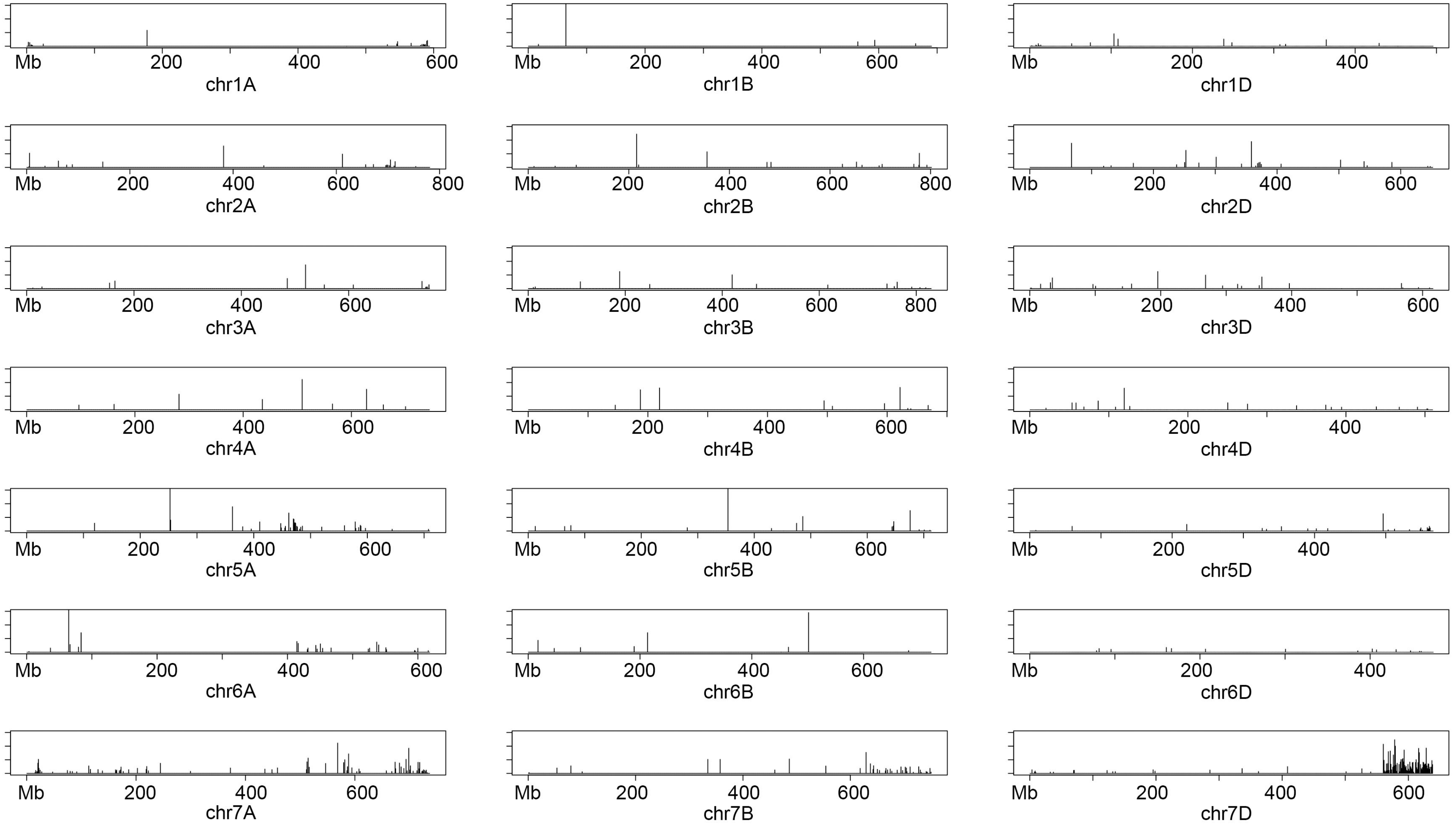

Supplementary Fig. 2: GBS plot of the *Sr43* wheat-*Th. elongatum* introgression EMS mutant 31b.

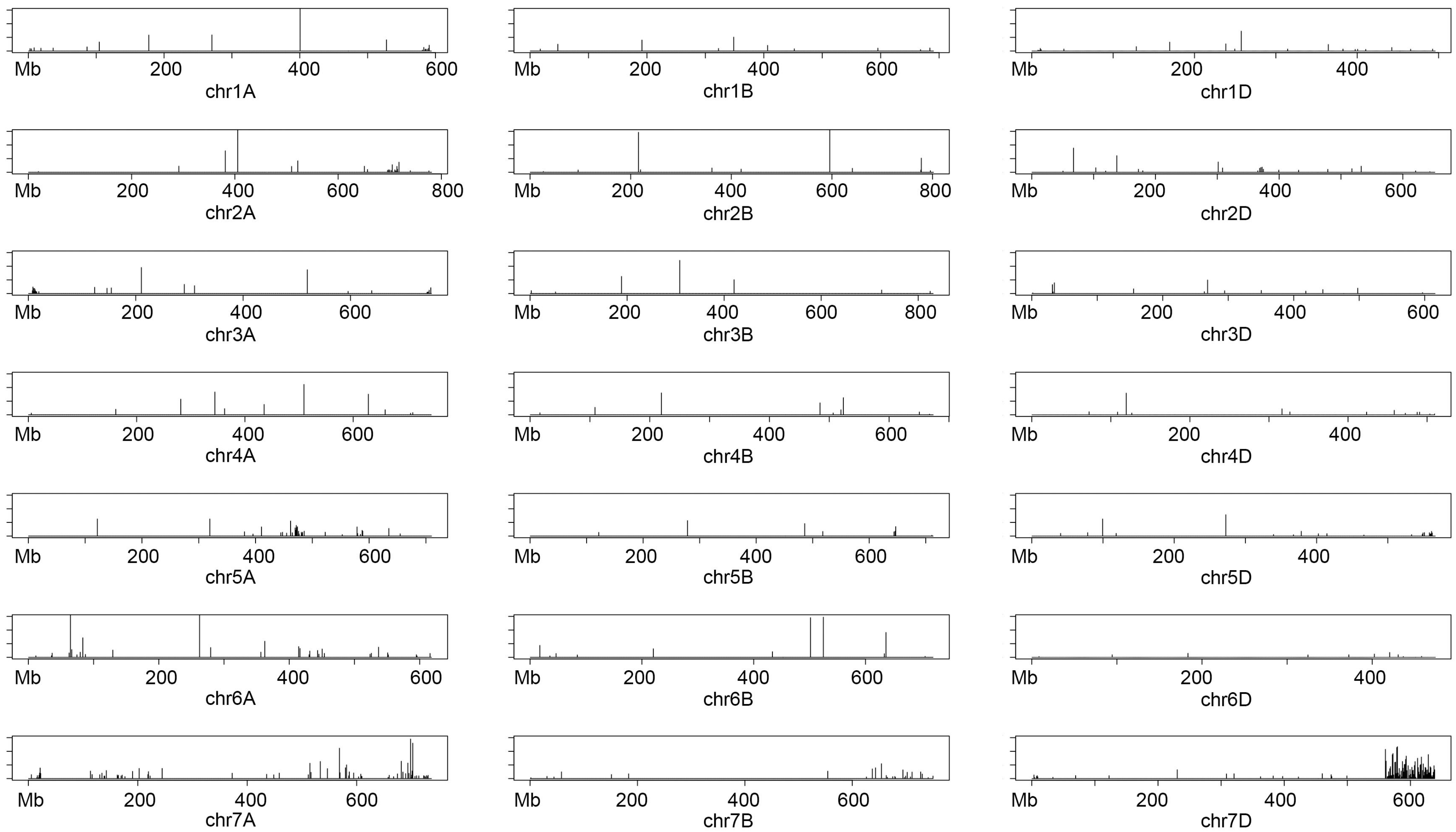

Supplementary Fig. 3: GBS plot of the *Sr43* wheat-*Th. elongatum* introgression EMS mutant 187g.

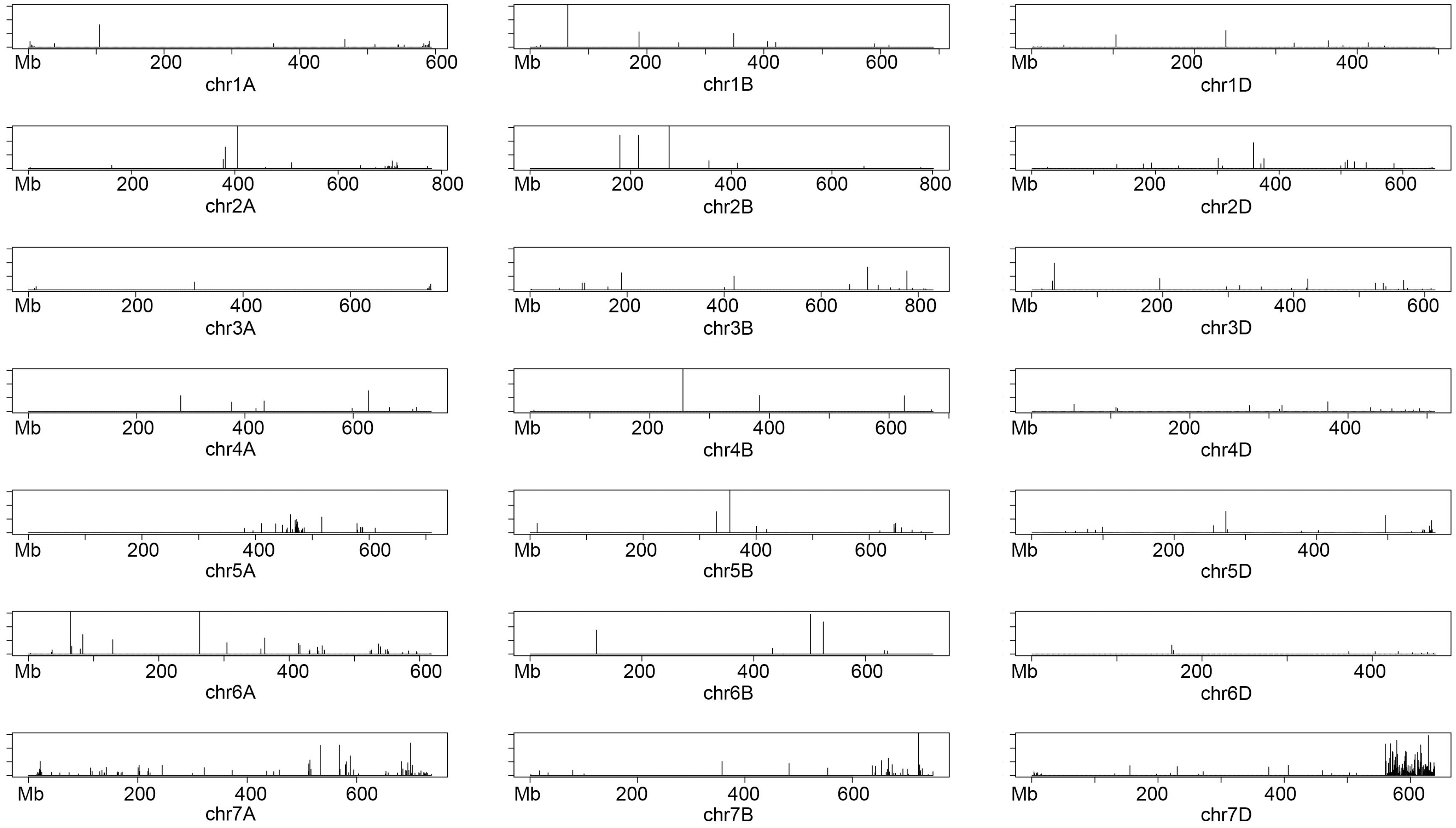

Supplementary Fig. 4: GBS plot of the *Sr43* wheat-*Th. elongatum* introgression EMS mutant 276b.

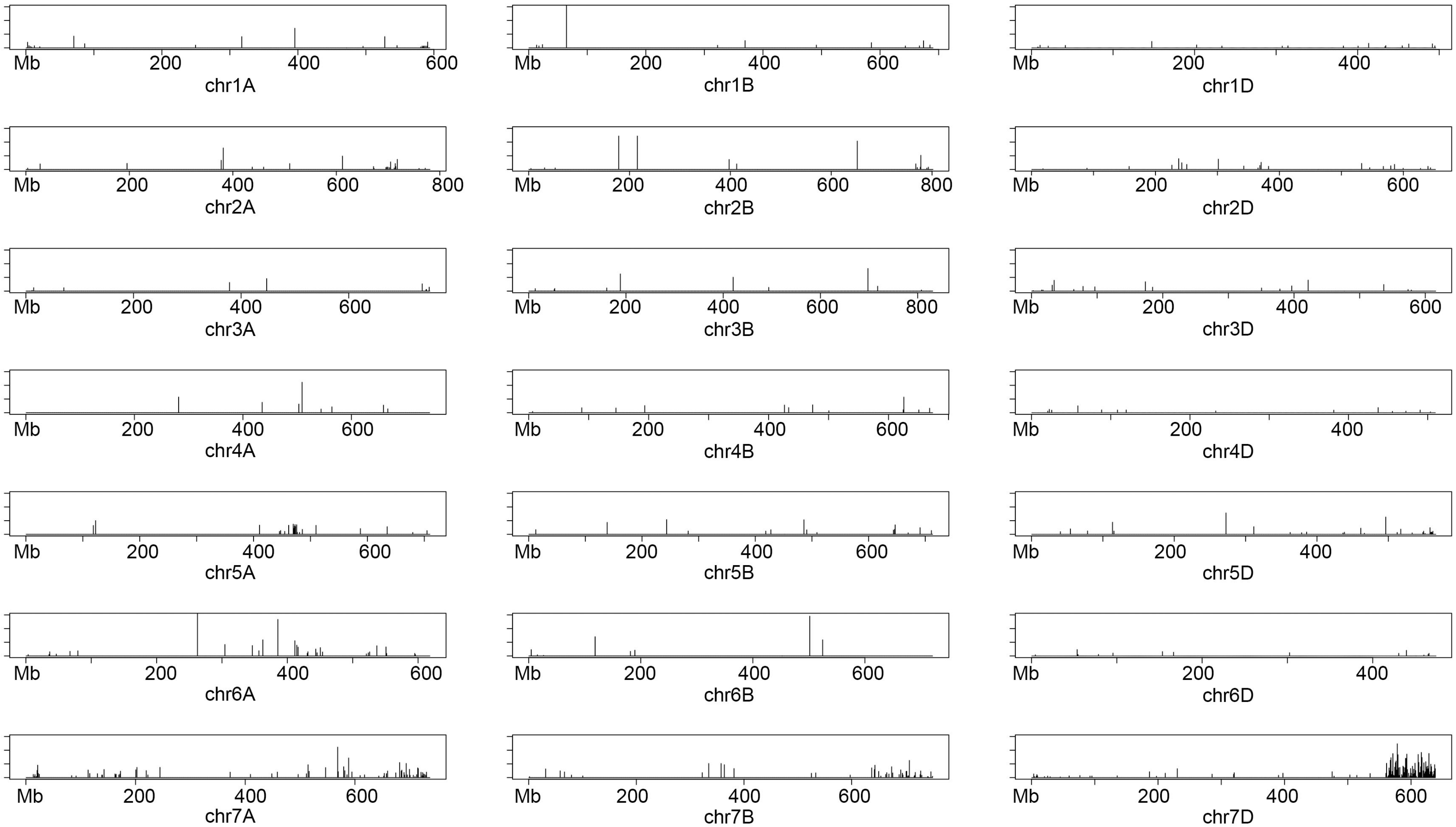

Supplementary Fig. 5: GBS plot of the *Sr43* wheat-*Th. elongatum* introgression EMS mutant 450b.

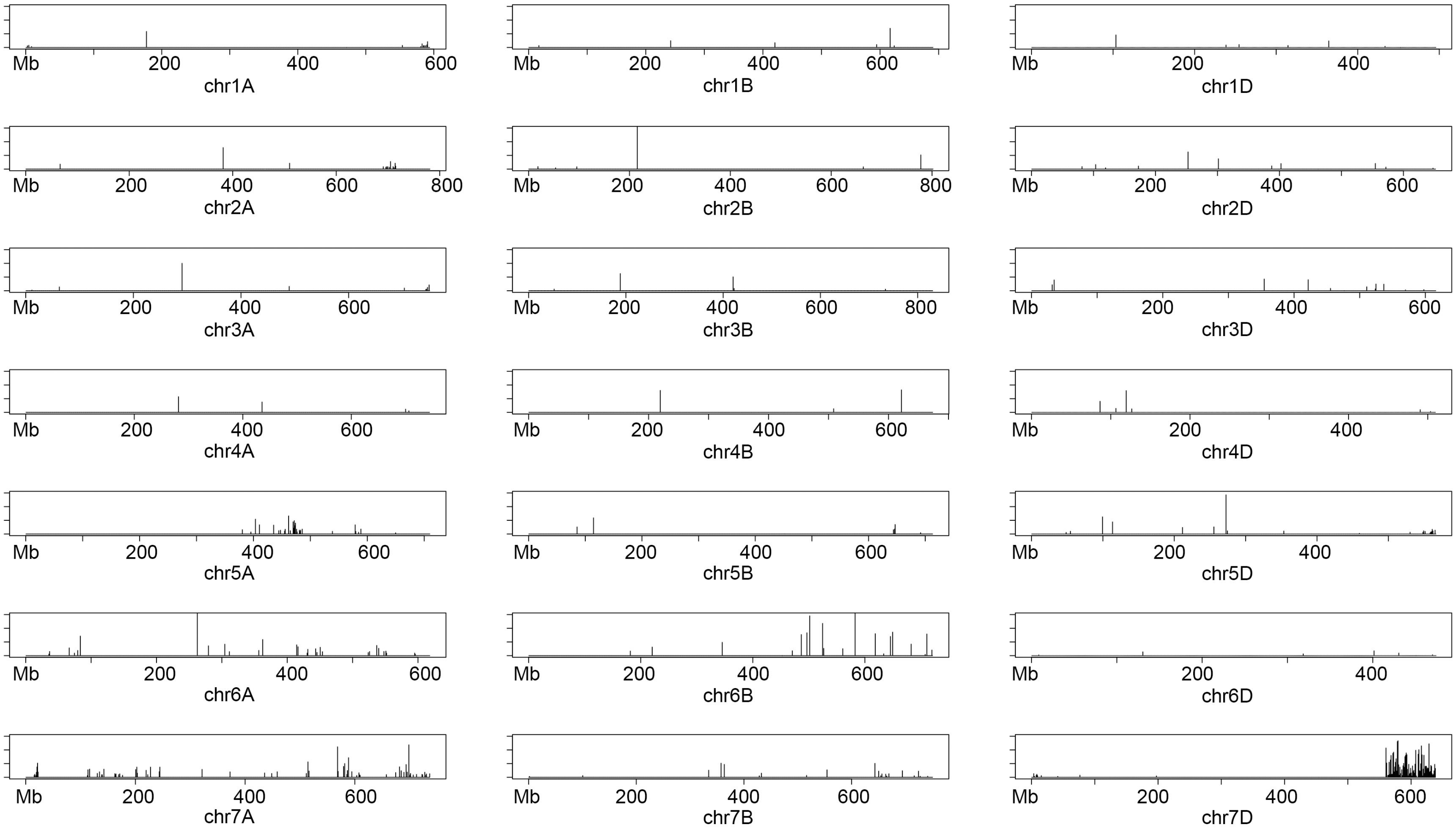

Supplementary Fig. 6: GBS plot of the *Sr43* wheat-*Th. elongatum* introgression EMS mutant 706f.

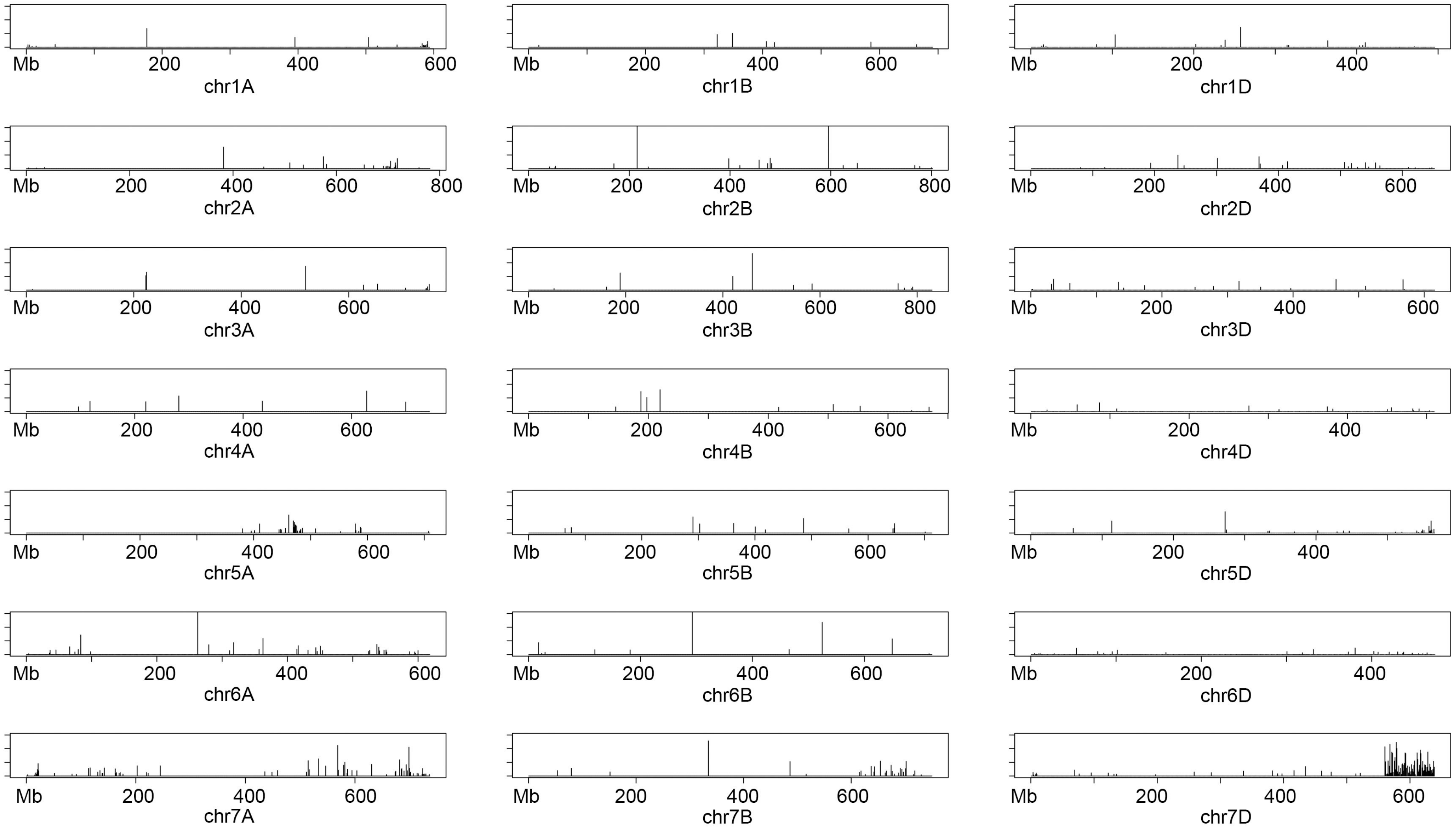

Supplementary Fig. 7: GBS plot of the *Sr43* wheat-*Th. elongatum* introgression EMS mutant 1013a.

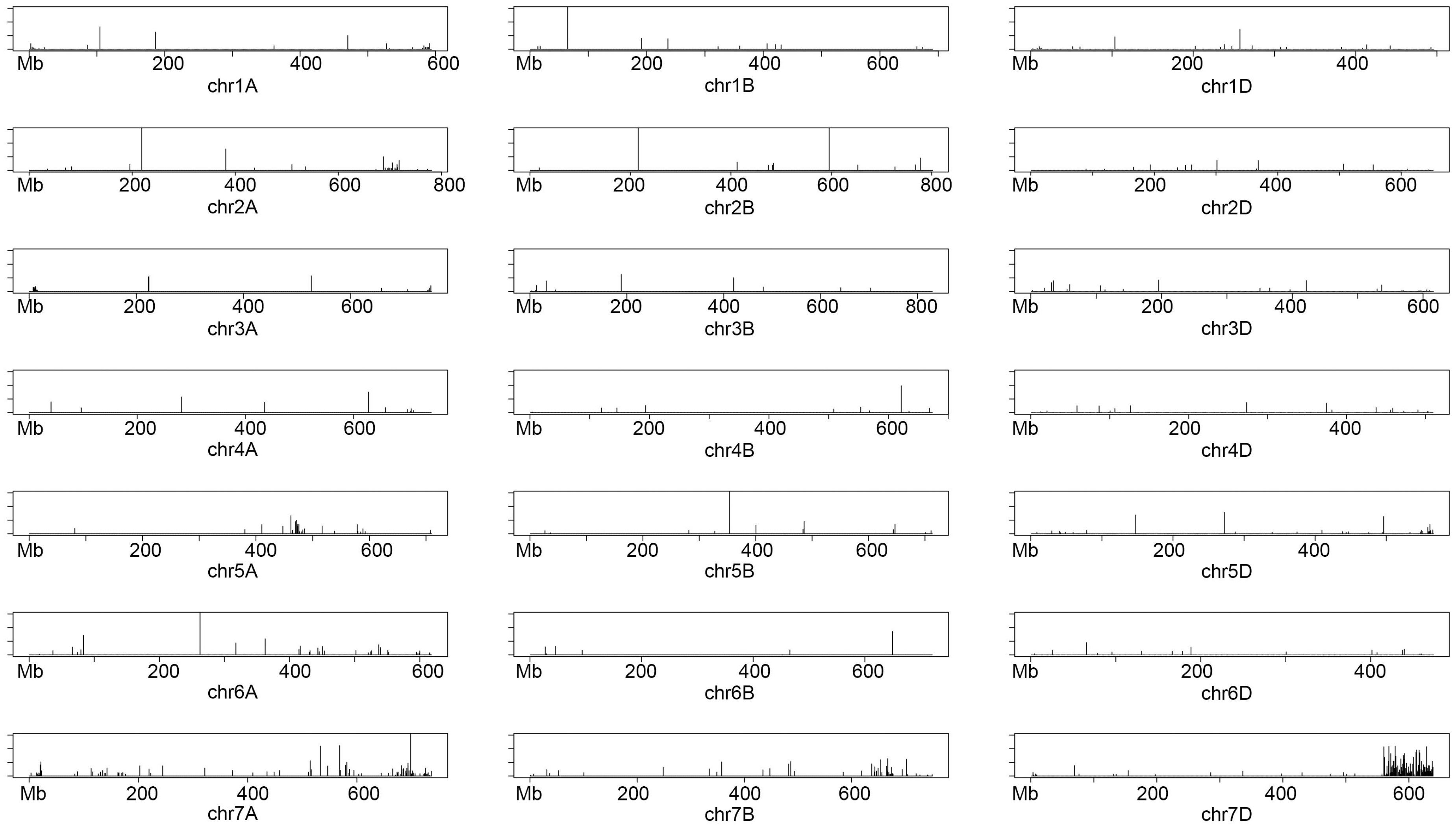

Supplementary Fig. 8: GBS plot of the *Sr43* wheat-*Th. elongatum* introgression EMS mutant 1102h.

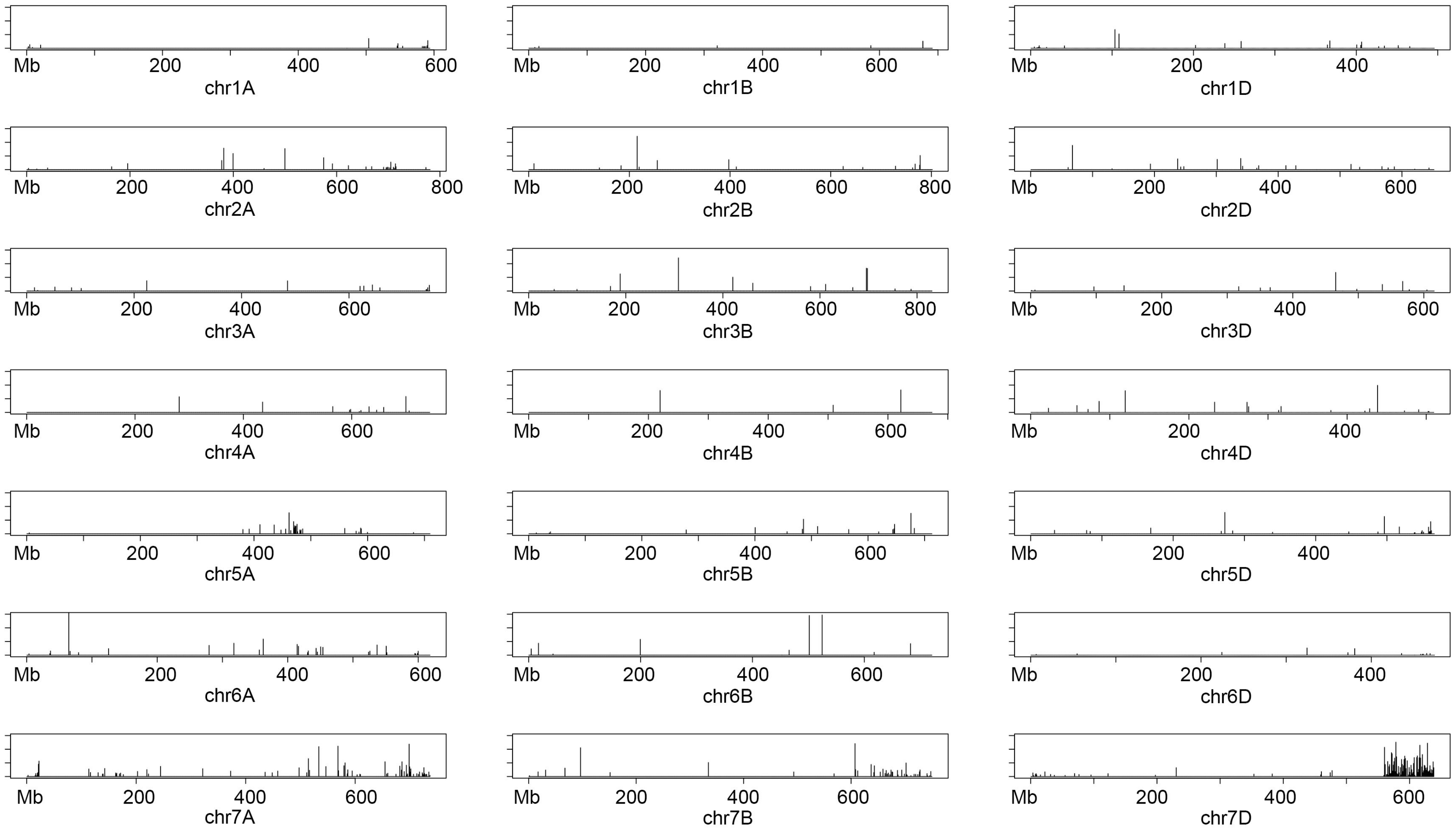

Supplementary Fig. 9: GBS plot of the *Sr43* wheat-*Th. elongatum* introgression EMS mutant 1298g.

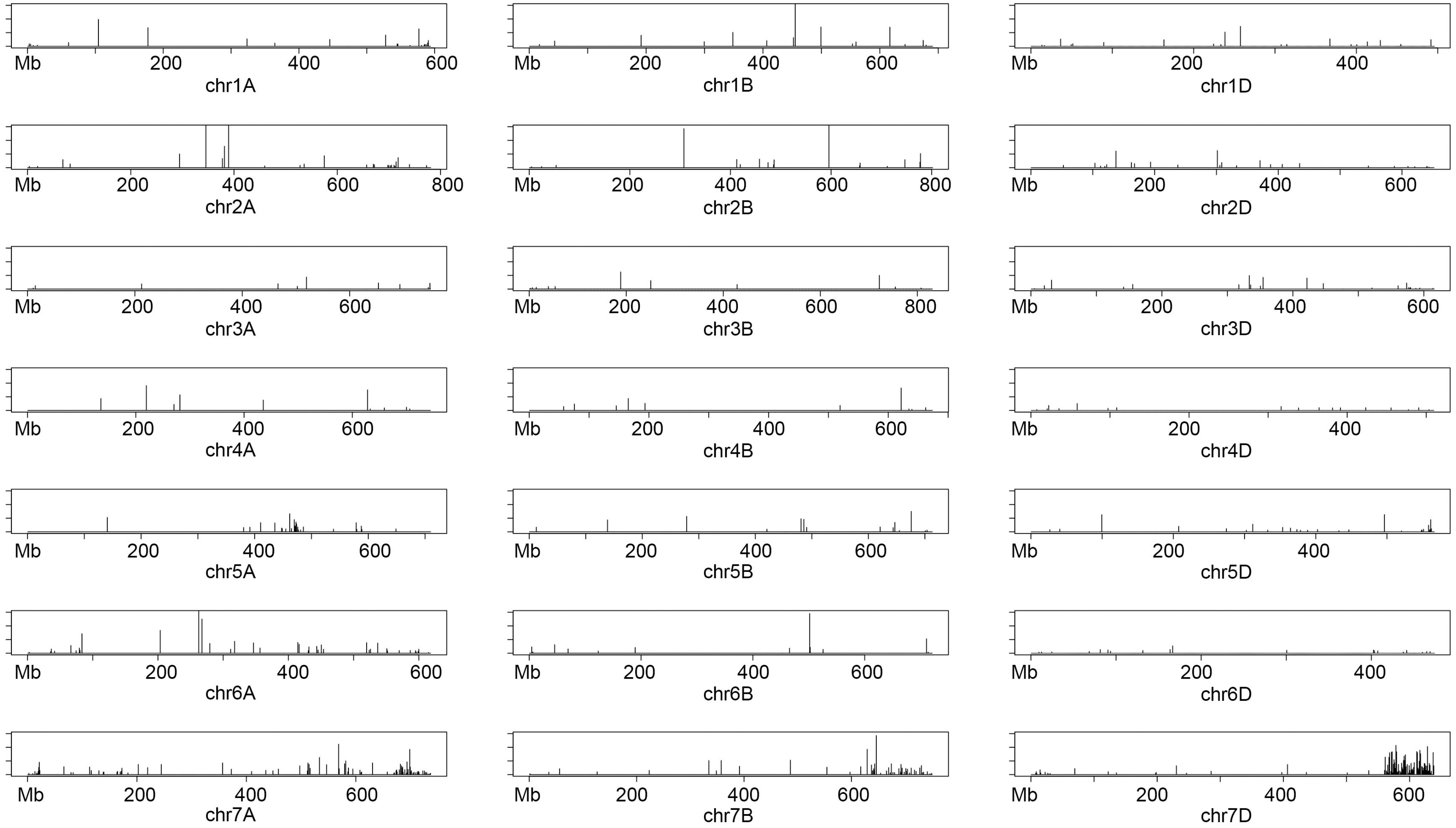

Supplementary Fig. 10: GBS plot of the *Sr43* wheat-*Th. elongatum* introgression EMS mutant 1513d.

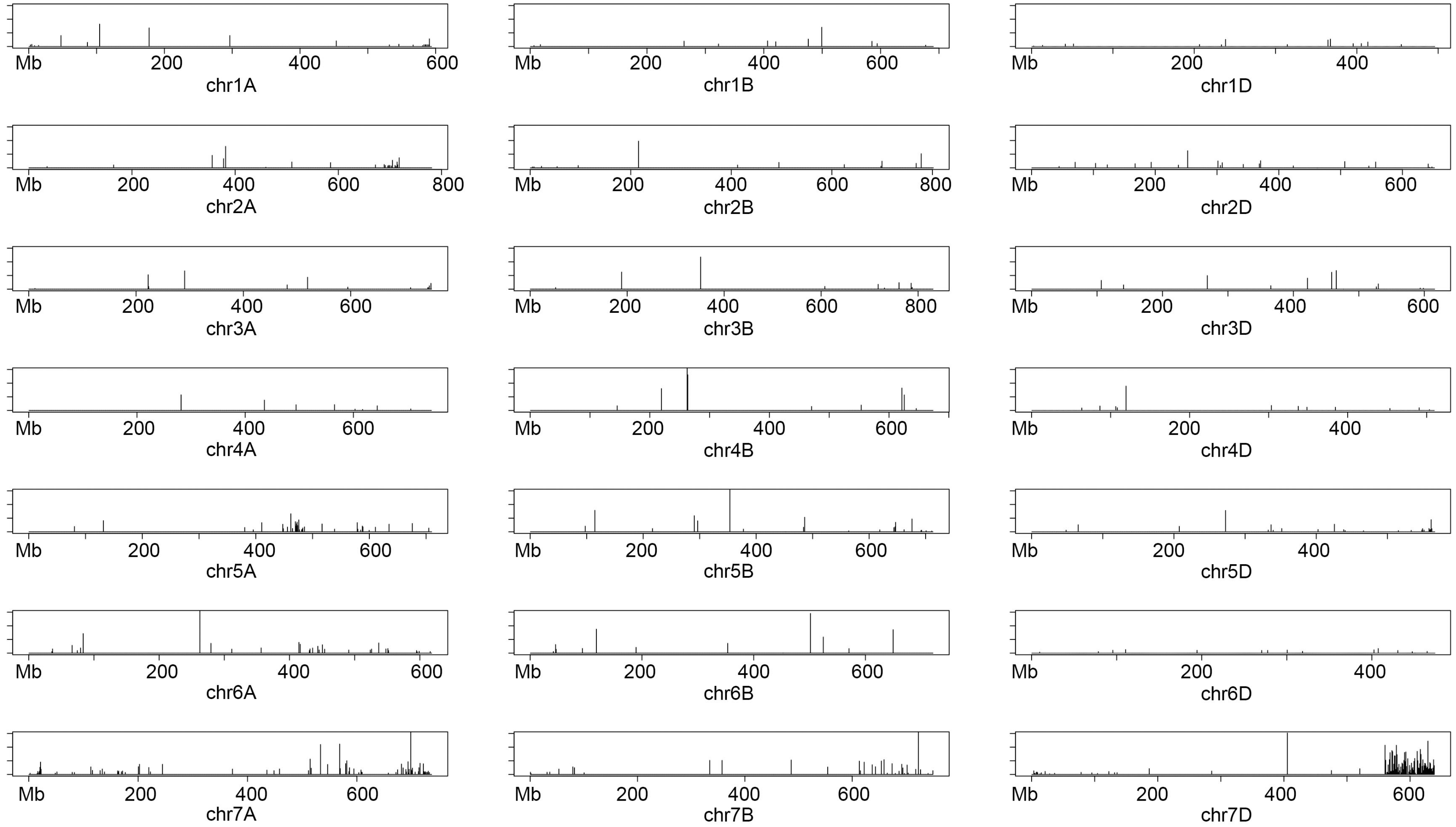

Supplementary Fig. 11: GBS plot of the *Sr43* wheat-*Th. elongatum* introgression EMS mutant 1671a.

```
Sr43            1 SYHLLGEITNGFSE------NRKLGTGAYGSVYKGEHKDGEKIAVKMLHD     44
                  |::.|..:||.|.|      ..|:|.|.:|.|||| :.:...:|||.|..
STKc_IRAK       1 SFYELKNVTNNFDERPISVGGNKMGEGGFGVVYKG-YVNNTTVAVKKLAA     49

Sr43           45 TLGLDNE----QFEKEYFNLADLQHKNIVRLVGYCHETRRECVPYNGRMV     90
                  .:.:..|    ||::|...:|..||:|:|.|:|:..:....|
STKc_IRAK      50 MVDITTEELKQQFDQEIKVMAKCQHENLVELLGFSSDGDDLC--------     91

Sr43           91 FADITKRALCFEYMRNGG-LDK--CLSDETSGHDWCTRYSIIKGICEGLK    137
                          |.:.||.||. ||:  || |.|....|..|..|.:|...|:.
STKc_IRAK      92 --------LVYVYMPNGSLLDRLSCL-DGTPPLSWHMRCKIAQGAANGIN    132

Sr43          138 YLHEELESPMYHLDLKPANILLDEKMVPKIADFGLSRFF-RGEQSQITKS    186
                  :||   |:...|.|:|.|||||||....||:||||:|.. :..|:.:|..
STKc_IRAK     133 FLH---ENHHIHRDIKSANILLDEAFTAKISDFGLARASEKFAQTVMTSR    179

Sr43          187 AIGTHGYVPPEYIDASVLSIKFDIFSLGVVIIKIMTGPTGYFRSAE----    232
                  .:||..|:.||.:...: :.|.||:|.|||:::|:||........|
STKc_IRAK     180 IVGTTAYMAPEALRGEI-TPKSDIYSFGVVLLEIITGLPAVDEHREPQLL    228

Sr43          233 MSAKQFIELVLANWRKRLPATSVYLLESY---------SEQVKRCTAIAV    273
                  :..|:.||            .....:|.|         |..|:...::|.
STKc_IRAK     229 LDIKEEIE------------DEEKTIEDYIDKKMNDADSTSVEAMYSVAS    266

Sr43          274 SCVEADRSKRPSIGEIVNKLNETETM    299
                  .|:...::|||.|.::...|.|....
STKc_IRAK     267 QCLHEKKNKRPDIKKVQQLLQEMTAS    292
```

Supplementary Fig. 12: Amino acid alignment between Sr43 and STKc IRAK. The identity is 30.4%, and the e-value is 2.06e-60.

```
                    52                  72
cAPK-α          1 LGTGSFGRVMLVKHMETGNHYAMKI------LDKQKVVK----LKQIEHT     40
                  ||||::|.|...:|.: |...|:|:      ||.::..|    |..::|
Sr43_kinase     1 LGTGAYGSVYKGEHKD-GEKIAVKMLHDTLGLDNEQFEKEYFNLADLQH-     48
                   91
cAPK-α         41 LNEKRILQAVNF---------PFLVKLEFSFKDNSNLYMVMEYVPGGEMF     81
                     |.|::.|.:         |:..::.|:  |.:...:..||:..|.:.
Sr43_kinase    49 ---KNIVRLVGYCHETRRECVPYNGRMVFA--DITKRALCFEYMRNGGLD     93
                                                              166
cAPK-α         82 SHLRRIGRFSEPHARFYAAQ------IVLTFEYLH---SLDLIYRDLKPE    122
                  ..|.     .|.....:..:      |....:|||   ...:.:.||||.
Sr43_kinase    94 KCLS-----DETSGHDWCTRYSIIKGICEGLKYLHEELESPMYHLDLKPA    138
                 171         184 186                      208
cAPK-α        123 NLLIDQQGYIQVTDFGFAKRVKGR----TWTLCGTPEYLAPEIILSKGYN    168
                  |:|:|::...::.|||.::..:|.    |.:..||..|:.||.|.:...:
Sr43_kinase   139 NILLDEKMVPKIADFGLSRFFRGEQSQITKSAIGTHGYVPPEYIDASVLS    188

cAPK-α        169 KAVDWWALGVLIYEMAAGYPPFF------ADQPIQIYEKIVSGKVRFPS-    211
                  ...|.::|||:|.::..|...:|      |.|.|::.  :.:.:.|.|:
Sr43_kinase   189 IKFDIFSLGVVIIKIMTGPTGYFRSAEMSAKQFIELV--LANWRKRLPAT    236
                                              280
cAPK-α        212 ------HFSSDLK---DLLRNLLQVDLTKR--FGNLKDGVNDIK     244
                        .:|..:|   .:..:.::.|.:||  .|.:.:.:|:.:
Sr43_kinase   237 SVYLLESYSEQVKRCTAIAVSCVEADRSKRPSIGEIVNKLNETE     280
```

Supplementary Fig. 13: Amino acid alignment between Sr43 and cAPK-α (Hanks, et al. 1988, DOI: 10.1126/science.3291115). Eight out of nine key amino acids are conserved in the Sr43 kinase. The numbers in red are the positions of nine key conserved amino acids of cAPK-α**.**

a

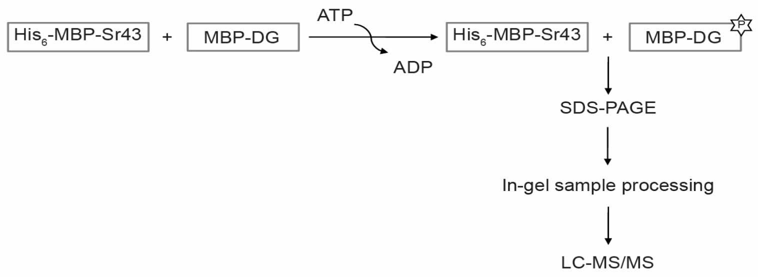

b

MASCOT Search Results

Protein View: Maltose_binding_protein_Recombinant_Substrate

sp|P00015|Maltose_binding_protein_Recombinant_Substrate

**Database:**	SR43
**Score:**	57069
**Monoisotopic mass (M_r):**	42456
**Calculated pI:**	5.08

Sequence similarity is available as an NCBI BLAST search of Maltose_binding_protein_Recombinant_Substrate against nr.

Search parameters

**MS data file:**	\\datawaha\corelabs\bioscience\bclp\ProtUsers\HHirt\Nag\2022\20221114_RFS1002207580.
**Enzyme:**	Trypsin/P: cuts C-term side of KR.
**Fixed modifications:**	Carbamidomethyl (C)
**Variable modifications:**	Oxidation (M), Phospho (ST), Phospho (Y)

Protein sequence coverage: 91%

Matched peptides shown in ***bold red***.

```
  1 MKIEEGKLVI WINGDKGYNG LAEVGKKFEK DTGIKVTVEH PDKLEEKFPQ
 51 VAATGDGPDI IFWAHDRFGG YAQSGLLAEI TPDKAFQDKL YPFTWDAVRY
101 NGKLIAYPIA VEALSLIYNK DLLPNPPKTW EEIPALDKEL KAKGKSALMF
151 NLQEPYFTWP LIAADGGYAF KYENGKYDIK DVGVDNAGAK AGLTFLVDLI
201 KNKHMNADTD YSIAEAAFNK GETAMTINGP WAWSNIDTSK VNYGVTVLPT
251 FKGQPSKPFV GVLSAGINAA SPNKELAKEF LENYLLTDEG LEAVNKDKPL
301 GAVALKSYEE ELAKDPRIAA TMENAQKGEI MPNIPQMSAF WYAVRTAVIN
351 AASGRQTVDE ALKDAQTNSS SNNNNNNNNN NLGIEGR
```

c

MASCOT Search Results

Protein View: Maltose_binding_protein_Recombinant_Substrate

sp|P00015|Maltose_binding_protein_Recombinant_Substrate

**Database:**	SR43
**Score:**	54530
**Monoisotopic mass (M_r):**	42456
**Calculated pI:**	5.08

Sequence similarity is available as an NCBI BLAST search of Maltose_binding_protein_Recombinant_Substrate against nr.

Search parameters

**MS data file:**	\\datawaha\corelabs\bioscience\bclp\ProtUsers\HHirt\Nag\2022\20221114_RFS1002207580.
**Enzyme:**	Trypsin/P: cuts C-term side of KR.
**Fixed modifications:**	Carbamidomethyl (C)
**Variable modifications:**	Oxidation (M), Phospho (ST), Phospho (Y)

Protein sequence coverage: 97%

Matched peptides shown in ***bold red***.

```
  1 MKIEEGKLVI WINGDKGYNG LAEVGKKFEK DTGIKVTVEH PDKLEEKFPQ
 51 VAATGDGPDI IFWAHDRFGG YAQSGLLAEI TPDKAFQDKL YPFTWDAVRY
101 NGKLIAYPIA VEALSLIYNK DLLPNPPKTW EEIPALDKEL KAKGKSALMF
151 NLQEPYFTWP LIAADGGYAF KYENGKYDIK DVGVDNAGAK AGLTFLVDLI
201 KNKHMNADTD YSIAEAAFNK GETAMTINGP WAWSNIDTSK VNYGVTVLPT
251 FKGQPSKPFV GVLSAGINAA SPNKELAKEF LENYLLTDEG LEAVNKDKPL
301 GAVALKSYEE ELAKDPRIAA TMENAQKGEI MPNIPQMSAF WYAVRTAVIN
351 AASGRQTVDE ALKDAQTNSS SNNNNNNNNN NLGIEGR
```

Supplementary Fig. 14. Schematic representation of the phosphorylation reaction and MASCOT search results. a, Schematic representation of the phosphorylation reaction and the sample preparation for LC-MS/MS analyses. **b**. Protein sequence coverage of maltose binding protein alone in the absence of His6-MBP-Sr43. **c**, Protein sequence coverage of maltose binding protein alone in the presence of His6-MBP-Sr43.

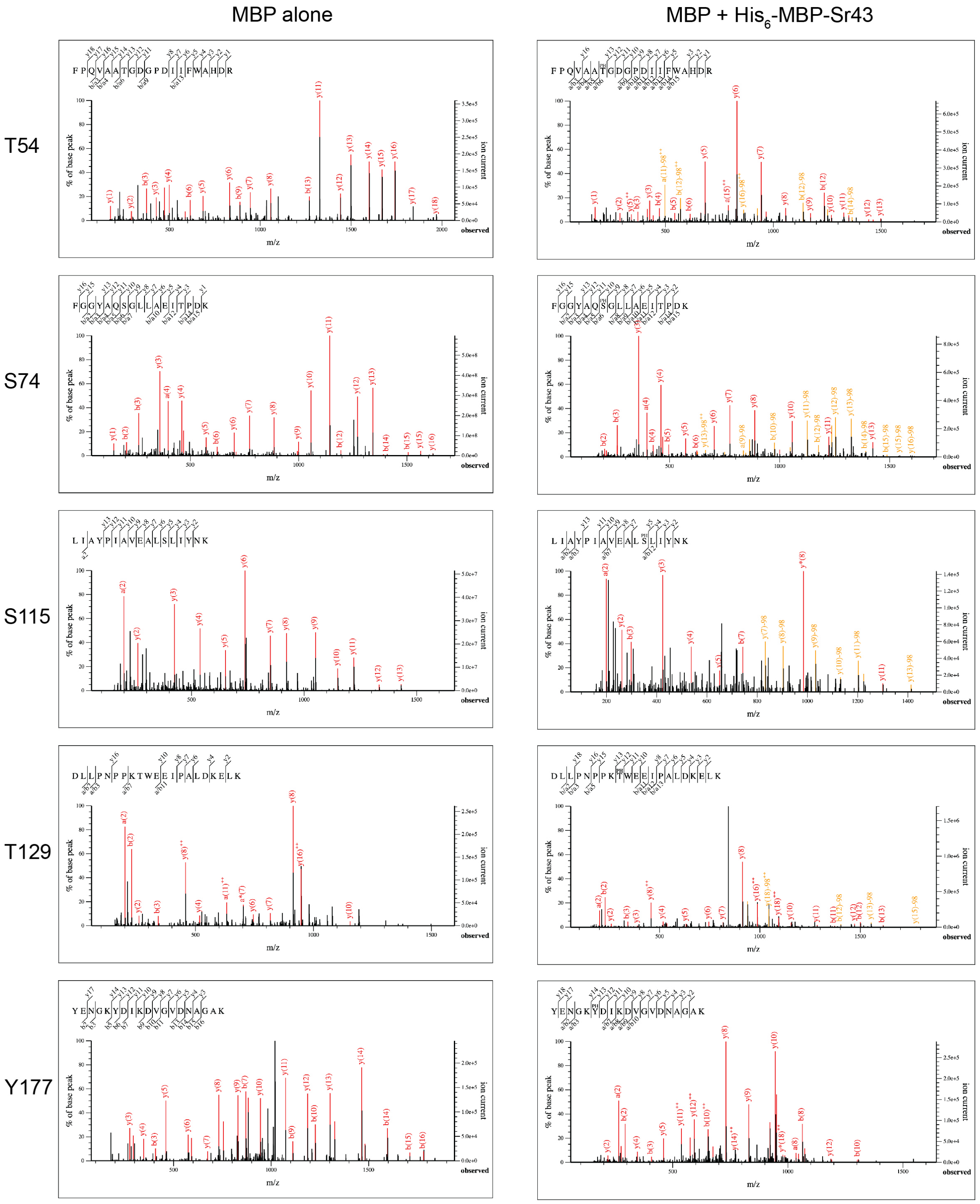

Supplementary Fig. 15. **Mass spectra of the peptides that are targeted by His6-MBP-Sr43 for phosphorylation of residues T54, S74, S115, T129 and Y177**. The amino acid numbers are indicated on the left. The spectra on the left panel correspond to the peptides identified from the sample without the kinase added in the reaction. The spectra on the right correspond to the same peptides but phosphorylated on the amino acid.

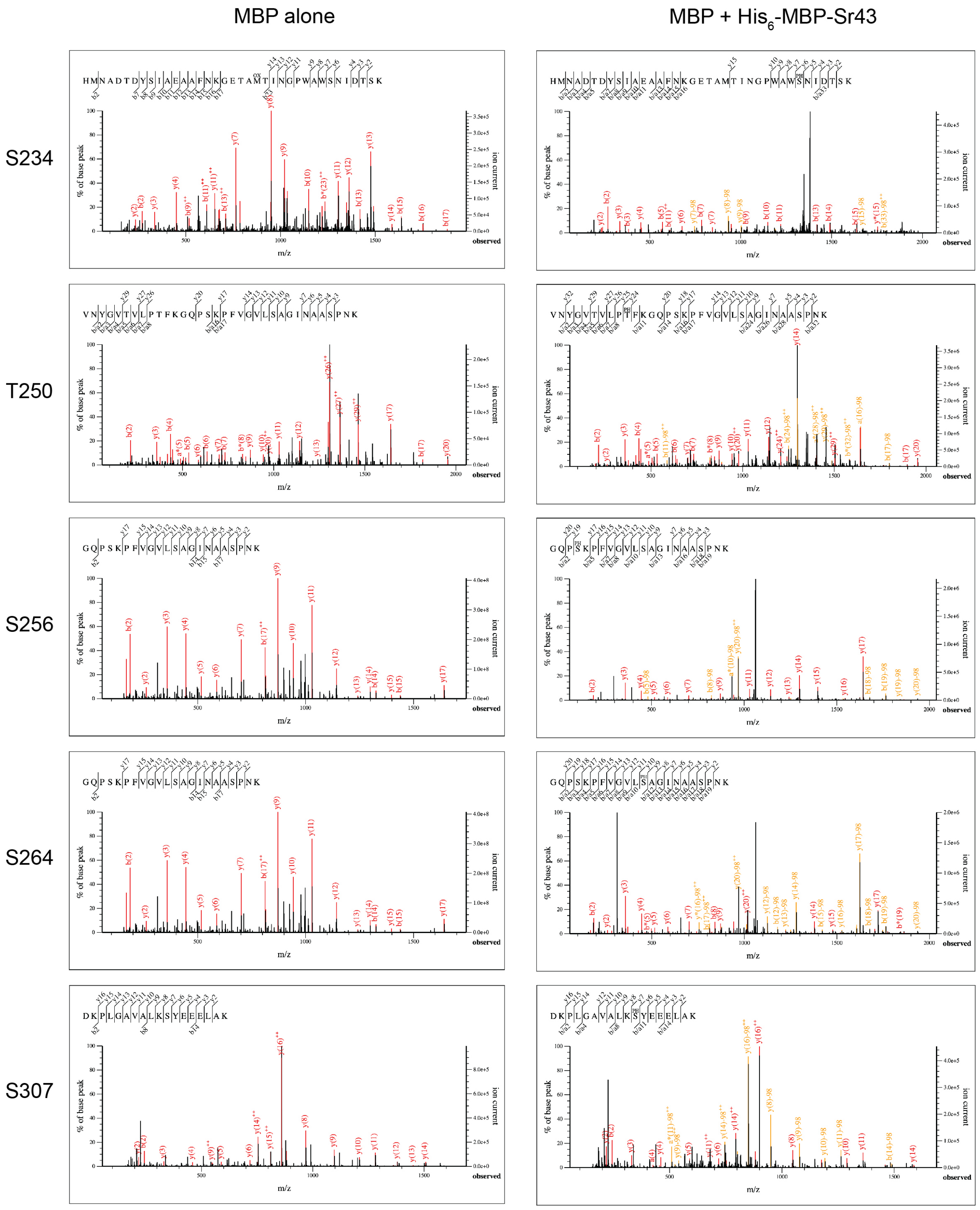

Supplementary Fig. 16. Mass spectra of the peptides that are targeted by His6-MBP-Sr43 for phosphorylation of residues S234, T250, S256, S264 and S307. The amino acid numbers are indicated on the left. The spectra on the left panel correspond to the peptides identified from the sample without the kinase added in the reaction. The spectra on the right correspond to the same peptides but phosphorylated on the amino acid.

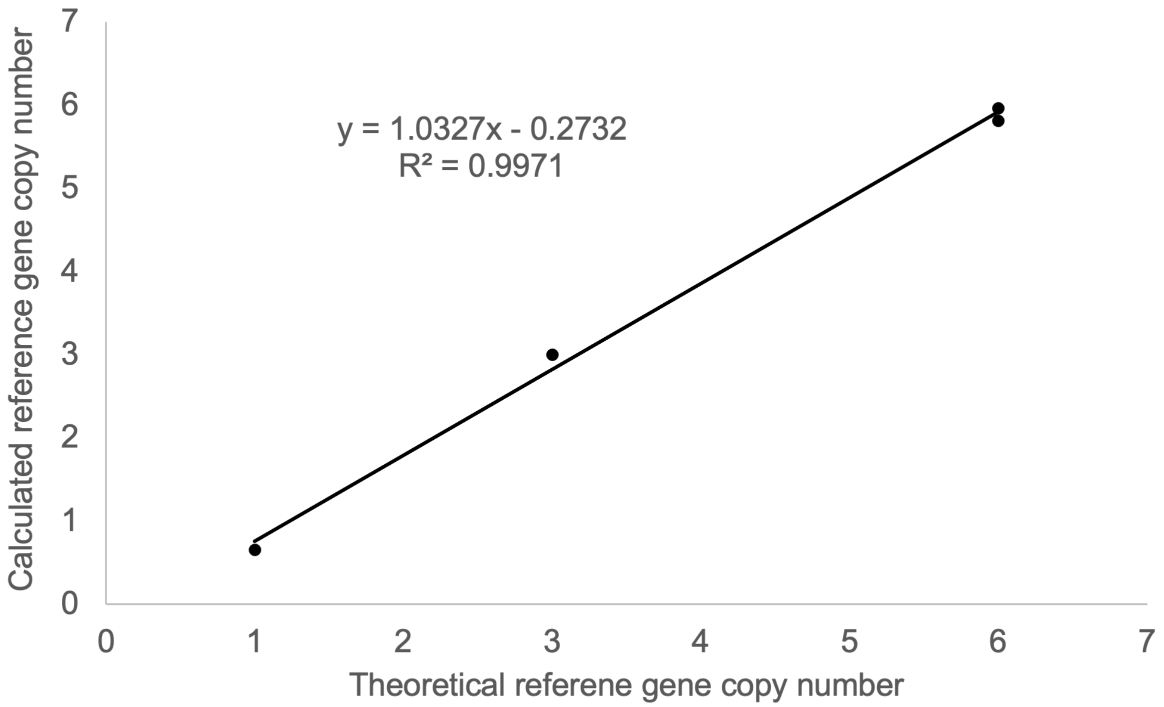

Supplementary Fig. 17. Regression of calculated reference gene copy number on theoretical reference gene copy number.
