# Supplementary material for: The wheat stem rust resistance gene Sr43 encodes an unusual protein kinase
Source: Nat Genet. 2023 May 22;55(6):921–6. doi: 10.1038/s41588-023-01402-1 (PMC10260397; doi:10.1038/s41588-023-01402-1)
Supplement: Supplementary file 2 — Reporting Summary [file 41588_2023_1402_MOESM2_ESM.pdf]

nature research

Corresponding author(s): Brande B. H. Wulff
Brian J. Steffenson

Last updated by author(s): 12 March 2023

Reporting Summary

Nature Research wishes to improve the reproducibility of the work that we publish. This form provides structure for consistency and transparency in reporting. For further information on Nature Research policies, see our Editorial Policies and the Editorial Policy Checklist.

Statistics

For all statistical analyses, confirm that the following items are present in the figure legend, table legend, main text, or Methods section.

n/a	Confirmed	
☐	☒	The exact sample size (*n*) for each experimental group/condition, given as a discrete number and unit of measurement
☒	☐	A statement on whether measurements were taken from distinct samples or whether the same sample was measured repeatedly
☒	☐	The statistical test(s) used AND whether they are one- or two-sided<br>*Only common tests should be described solely by name; describe more complex techniques in the Methods section.*
☒	☐	A description of all covariates tested
☐	☒	A description of any assumptions or corrections, such as tests of normality and adjustment for multiple comparisons
☒	☐	A full description of the statistical parameters including central tendency (e.g. means) or other basic estimates (e.g. regression coefficient) AND variation (e.g. standard deviation) or associated estimates of uncertainty (e.g. confidence intervals)
☒	☐	For null hypothesis testing, the test statistic (e.g. *F*, *t*, *r*) with confidence intervals, effect sizes, degrees of freedom and *P* value noted<br>*Give P values as exact values whenever suitable.*
☒	☐	For Bayesian analysis, information on the choice of priors and Markov chain Monte Carlo settings
☒	☐	For hierarchical and complex designs, identification of the appropriate level for tests and full reporting of outcomes
☒	☐	Estimates of effect sizes (e.g. Cohen's *d*, Pearson's *r*), indicating how they were calculated

Our web collection on statistics for biologists contains articles on many of the points above.

Software and code

Policy information about availability of computer code

Data collection

No software was used to collect the stem rust phenotype data.

Data analysis

Assembly of Sr43 chromosome:
Meraculous v 2.0.5

Mutant mapping:
BWA v. 0.7.12
SAMtools v1.8

GBS plotting:
bwa mem v0.7.12 (default parameters)
SAMtools v.0.1.19
Custom scripts: https://github.com/steuernb/GBS_introgression_line_analysis linked to Zenodo via https://zenodo.org/badge/latestdoi/394326594

RNA mapping:
Trimmomatic v.
Hisat2 v. 2.1.0
Samtools v. 1.8
IGV v.

Annotation of Sr43:
InterPro v. 88.0

Myristoylator: Bologna G., et al. N-terminal myristoylation predictions by ensembles of neural networks. Proteomics. 4,1626–1632 (2004).
NLS mapper: Kosugi, S. et al. Systematic identification of yeast cell cycle-dependent nucleocytoplasmic shuttling proteins by prediction of composite motifs. Proc. Natl. Acad. Sci. U.S.A. 106, 10171–10176 (2009).

Phylogenetic tree construction:
https://www.ebi.ac.uk/Tools/msa/clustalo/
https://itol.embl.de/
Protein scan: hmmscan v3.1b2

3D modeling, structure comparison and docking:
AlphaFold v2.0 (https://alphafold.ebi.ac.uk)
Dali (http://ekhidna2.biocenter.helsinki.fi/dali/)
HADDOCK2.4 (https://www.bonvinlab.org/education/HADDOCK-binding-sites/.

For manuscripts utilizing custom algorithms or software that are central to the research but not yet described in published literature, software must be made available to editors and reviewers. We strongly encourage code deposition in a community repository (e.g. GitHub). See the Nature Research guidelines for submitting code & software for further information.

Data

Policy information about availability of data

All manuscripts must include a data availability statement. This statement should provide the following information, where applicable:

- Accession codes, unique identifiers, or web links for publicly available datasets
- A list of figures that have associated raw data
- A description of any restrictions on data availability

The datasets generated during and/or analyzed in the current study are publicly available as follows. The sequence reads were deposited in the European Nucleotide Archive under project numbers PRJEB52878 (GBS data), PRJEB51958 (chromosome flow sorted data), and PRJEB52088 (RNA-seq data). The Sr43 gene and transcript sequence were deposited in NCBI Genbank under accession number ON237711. The Sr43 chromosome assembly has been deposited in Zenodo with DOI 10.5281/zenodo.6777941. The following public databases/datasets were used in the study: Chinese Spring reference genome39, Gramene (http://www.gramene.org/), https://ensembl.gramene.org/Multi/Tools/Blast, https://wheat.pw.usda.gov/GG3/blast, BLAST non-redundant protein sequence (https://blast.ncbi.nlm.nih.gov/Blast.cgi?PROGRAM=blastx&PAGE_TYPE=BlastSearch&LINK_LOC=blasthome), Taxonomy Browser (https://www.ncbi.nlm.nih.gov/Taxonomy/Browser/wwwtax.cgi?id=1437183), AlphaFold27 (https://alphafold.ebi.ac.uk), Dali28 (http://ekhidna2.biocenter.helsinki.fi/dali/), and HADDOCK25 (https://www.bonvinlab.org/education/HADDOCK-binding-sites/. Source data are provided with this paper.

The following public databases/datasets were used in the study:

Chinese Spring reference genome (IWGSC, 2018)
Gramene: http://www.gramene.org/#
BLAST https://blast.ncbi.nlm.nih.gov/Blast.cgi?PROGRAM=blastx&PAGE_TYPE=BlastSearch&LINK_LOC=blasthome
(non-redundant protein sequence-nr)
Taxonomy Browser: https://www.ncbi.nlm.nih.gov/Taxonomy/Browser/wwwtax.cgi?id=1437183
AlphaFold v2.0 (https://alphafold.ebi.ac.uk)
Dali (http://ekhidna2.biocenter.helsinki.fi/dali/)
HADDOCK 2.4 (https://www.bonvinlab.org/education/HADDOCK-binding-sites/.

Source data are provided with this paper.

Field-specific reporting

Please select the one below that is the best fit for your research. If you are not sure, read the appropriate sections before making your selection.

☒ Life sciences ☐ Behavioural & social sciences ☐ Ecological, evolutionary & environmental sciences

For a reference copy of the document with all sections, see nature.com/documents/nr-reporting-summary-flat.pdf

Life sciences study design

All studies must disclose on these points even when the disclosure is negative.

Sample size	No sample size calculation was chosen.
Data exclusions	We did not exclude any data points from the study.
Replication	Mutant and transgenic phenotypes were repeated once with similar outcomes
Randomization	The stem rust tests were not randomized.
Blinding	No blinding was applied.

Reporting for specific materials, systems and methods

We require information from authors about some types of materials, experimental systems and methods used in many studies. Here, indicate whether each material, system or method listed is relevant to your study. If you are not sure if a list item applies to your research, read the appropriate section before selecting a response.

Materials & experimental systems

n/a	Involved in the study
☒	☐ Antibodies
☒	☐ Eukaryotic cell lines
☒	☐ Palaeontology and archaeology
☒	☐ Animals and other organisms
☒	☐ Human research participants
☒	☐ Clinical data
☒	☐ Dual use research of concern

Methods

n/a	Involved in the study
☒	☐ ChIP-seq
☒	☐ Flow cytometry
☒	☐ MRI-based neuroimaging
